# Hearing loss, depression, and cognition in younger and older adult CI candidates

**DOI:** 10.3389/fneur.2023.1272210

**Published:** 2023-10-13

**Authors:** Maria Huber, Lisa Reuter, Lennart Weitgasser, Belinda Pletzer, Sebastian Rösch, Angelika Illg

**Affiliations:** ^1^Department of Otorhinolaryngology, Head and Neck Surgery, Paracelsus Medical University Salzburg, Salzburg, Austria; ^2^Clinic for Otorhinolaryngology, Medical University of Hannover, Hannover, Germany; ^3^Department of Psychology, Center for Neurocognitive Research, University of Salzburg, Salzburg, Austria

**Keywords:** hearing loss from adulthood, secondary depression, cognition, younger and older adults, hearing restrictions during communication in quiet and in noise

## Abstract

**Background and Aim:**

Hearing loss in old age is associated with cognitive decline and with depression. Our study aimed to investigate the relationship between hearing loss, cognitive decline, and secondary depressive symptoms in a sample of younger and older cochlear implant candidates with profound to severe hearing loss.

**Methods:**

This study is part of a larger cohort study designated to provide information on baseline data before CI. Sixty-one cochlear implant candidates with hearing loss from adulthood onwards (>18 years) were enrolled in this study. All had symmetrical sensorineural hearing loss in both ears (four-frequency hearing threshold difference of no more than 20 dB, PTA). Individuals with primary affective disorders, psychosis, below-average intelligence, poor German language skills, visual impairment, and a medical diagnosis with potential impact on cognition (e.g., neurodegenerative diseases,) were excluded. Four-frequency hearing thresholds (dB, PTA, better ear) were collected. Using the Abbreviated Profile of Hearing Aid Benefit, we assessed subjective hearing in noise. Clinical and subclinical depressive symptoms were assessed with the Beck Depression Inventory (BDI II). Cognitive status was assessed with a neurocognitive test battery.

**Results:**

Our findings revealed a significant negative association between subjective hearing in noise (APHAB subscale “Background Noise”) and BDII. However, we did not observe any link between hearing thresholds, depression, and cognition. Additionally, no differences emerged between younger (25–54 years) and older subjects (55–75 years). Unexpectedly, further unplanned analyses unveiled correlations between subjective hearing in quiet environments (APHAB) and cognitive performance [phonemic fluency (Regensburg Word Fluency), cognitive flexibility (TMTB), and nonverbal episodic memory (Nonverbal Learning Test), as well as subjective hearing of aversive/loud sounds (APHAB)], cognitive performance [semantic word fluency (RWT), and inhibition (Go/Nogo) and depression]. Duration of hearing loss and speech recognition at quiet (Freiburg Monosyllables) were not related to depression and cognitive performance.

**Conclusion:**

Impact of hearing loss on mood and cognition appears to be independent, suggesting a relationship with distinct aspects of hearing loss. These results underscore the importance of considering not only conventional audiometric measures like hearing thresholds but also variables related to hearing abilities during verbal communication in everyday life, both in quiet and noisy settings.

## Introduction

The impact of hearing loss is generally underestimated. The Global Burden of Disease study (1) reveals that hearing loss ranks as the third highest cause of disability globally in terms of years of life with disability, making it the foremost sensory impairment. Moreover, in the United States, hearing loss is the third most prevalent chronic physical condition, surpassing the occurrence of diabetes and cancer (2). Notably, it predominantly affects people aged 55 and older (1). In the United States, age-related lifetime hearing loss is estimated to impose an economic burden of approximately $297,000 per affected individual on society (3). Moreover, hearing loss is associated with cognitive decline (4–15).

Aging (16–18) is also associated with cognitive decline and increasing rates of dementia (19). and sensorineural hearing loss, especially if left untreated, appears to further accelerate cognitive decline. Significant associations were found between hearing thresholds (dB PTA) and cognitive performance [Retrospective: (6, 7, 9–14)]; Prospective: (5, 15, 20); Systematic review: (7, 8).^1^ Starting with a subclinical hearing loss < 25 dB, audiometric hearing loss (pure tone average) was associated with cognitive decline [Subclinical hearing loss (10), mild hearing loss (11, 12), moderate and severe hearing loss (5, 21)]. Further findings suggest that with increasing age, there is a progressively greater cognitive decline from older adults with hearing loss (14, 21). However, Croll et al. did not find that hearing loss is accelerating cognitive decline in non demented older adults (15).

Recently published studies have shed light on the significance of both peripheral hearing and central auditory components for cognitive performance. Among older adults, individuals with speech recognition scores in noise below the median exhibited poorer cognitive performance and experienced accelerated cognitive decline over an average follow-up period of 3.5 years compared to those with scores above the median (20). Furthermore, speech recognition in noise in healthy, mostly elderly Chinese was associated with both cognitive performance and parahippocampal cortex (medial temporal lobe) thickness. The parahippocampal cortex was identified as a partial mediator between hearing in noise and cognition, suggesting its potential role between the two (22).

Several articles discuss possible mechanism linking adult hearing loss in older adults and cognitive decline (3, 23–30). Causal mechanisms as well as shared (common) mechanisms have been proposed. An example of a hypothesis addressing common mechanisms (“common cause”) is that age-related functional and structural changes in the brain (spontaneous neural activity and functional brain connectivity, changes in brain areas)^2^ account for changes in auditory processes and cognitive processes in old age (24, 29). An example of a causal hypothesis is that hearing loss is the cause of social isolation that mediates cognitive decline [e.g., (24, 29, 34)]. Rutherford et al., Uchida et al., Slade et al., and Sharma et al. discussed the possible contribution of depression as a consequence of hearing loss to cognitive decline in older adults (25, 27, 28, 30).^3^

To date, there has been a lack of research investigating the potential mediating role of depression between hearing loss and cognitive performance.

In addition to hearing loss, depression itself appears to independently contribute to cognitive decline. Multiple studies have consistently demonstrated a link between depressive problems and cognitive decline (35–41). Moreover, not only the severity but also the age of onset of depressive symptoms has been found to have a negative impact on cognitive performance (35, 39, 40, 42). Notably, individuals with bipolar disorder tend to experience more cognitive difficulties than those with major depression (43). Executive functions such as attention, working memory, and cognitive flexibility are affected, as well as episodic memory and cognitive processing speed. Depression is also associated with changes in the brain including the limbic system (hippocampus, amygdala, cingulate gyrus, hypothalamus, insula), the prefrontal cortex, and the cerebellum (44–47).

Numerous recent retrospective (6, 48–57) and prospective (58) studies indicate a connection between reduced hearing and depression. Bilateral audiometric hearing loss was found to be associated with depression [Golub et al. ≥ 25 dB HL better ear, Golub et al. beginning with subclinical HL of >15 dB, Shukla et al. ≥ 25 dB HL, better ear; Chern et al. ≥ 25 dB HL, left ear and right ear (53–56)]. Additionally, it seems to increase the risk for depression, also after multivariate comparison (58). However, an older study found no association between audiometric hearing loss and depression (59).

It is important to note that the assessment of depression in various studies may vary, leading to potential confusion. Most authors (6, 48, 51, 53–57) employed validated depression questionnaires, such as the Epidemiological Studies Depression Scale-10 (60). Brewster et al. and Tsimpida et al. utilized a continuum of depressive symptoms, covering both clinical and subclinical manifestations (51, 58). On the other hand, Li et al., Golub et al., Brewster et al., Chern et al., and Shukla focused solely on clinical symptoms (48, 52–56), while Tseng et al. and Kim relied on psychiatric diagnoses of depressive disorders (49, 58).

In addition, several studies inform about associations between hearing loss, depression, and brain changes in middle-aged and older adults. Tang et al. reported that middle-aged adults with acquired sensorineural hearing loss had higher levels of depression and anxiety and lower functional connectivity between the amygdala and auditory cortex, striatum, multimodal processing areas, and fronto-parietal control areas compared with normal-hearing peers (61). Aoki et al. (62) found that moderate/severe hearing loss was associated with higher depression scores in older adults, hippocampal atrophy and a higher cortisol/dehydroepiandrosterone sulfate ratio, which indicates higher stress levels (62). Brewster et al. examined older people with untreated hearing loss and major depression according to DSM 5 (Diagnostic and Statistical Manual) (63, 64). Significant associations were found between lower speech recognition and cortical thinning in the primary and secondary auditory cortex and lower integrity of the superior longitudinal fasciculus.

There is also evidence that hearing and depression are related to each other via the neurotransmitter serotonin. Impaired serotonergic function in the auditory cortex has been found in normal-hearing adults with major depression (65). Studies in rodents show that hearing damage is associated with changes in the serotonergic system within the auditory system, but also in the hypothalamus, striatum, and frontal cortex” (66–68).

Our clinical experience and research by GBD 19 Hearing Loss Collaborators (1) confirm that hearing loss is associated with depression. Many adults with hearing loss struggle to follow conversations, especially in noisy environments or with multiple people speaking, leading to feelings of depression.

To date, only a limited number of studies have explored the potential link between depression resulting from hearing loss and cognitive decline in adults with hearing loss. One study (69) found that the cognitive decline in older adults with hearing loss, compared to normal hearing, was partially associated with the severity of depressive symptoms. Another study (70) observed that older adults with hearing loss and clinical depressive symptoms experienced greater cognitive decline over a 10-year period compared to those without depression. However, the question of whether there is a direct relationship between depression as a result of hearing loss and cognitive performance, as hypothesized in the models of Rutherford et al., Uchida et al., Sharma et al. (see above), has not yet been investigated (25, 27, 30).

Taken together, the studies published to date support the hypotheses that there are associations between adult hearing loss (peripheral and central hearing) and depression and cognitive performance, and that depression mediates between hearing loss and cognitive performance. However, some limitations of these studies should also be considered. Brewster et al. (51) and Tsimpida et al. (57) used exclusively self-reports single questions to assess hearing loss, for example “Can you hear well enough to carry on a conversation in a crowded room?” (51). Hearing loss is usually defined as a four-frequency PTA of >25 dB HL on the better ear^4^ [see the website of US National Institute of Deafness and other Communication Disorders (71)]. Study results indicate that the exclusive use of self-report single questions is not sufficient to detect such a defined hearing loss (72–75). None of the studies, with the exception of Tseng et al. distinguished between primary and secondary depression, i.e., depression as a presumed consequence of hearing loss (58). In addition, several studies used verbal tasks, which may have confounded the results^5^ (10, 12, 13, 15, 20, 70). In addition, only some of the studies excluded individuals with suspected cognitive impairment or dementia (9, 12, 13, 15, 69, 76). Very few authors (13, 15, 58, 69) provide information on the time of onset of hearing loss (e.g., congenital hearing loss, hearing loss in adulthood). Finally, it is important to remember that hearing loss can affect not only older adults but also younger adults (1). To date, however, the majority of studies have been conducted in older adults, with an average age greater than 65 years [studies conducted with younger adults with a mean age less than 65 years include (5, 9, 48, 49, 58)]. It is not known, whether cognitive decline also occurs in younger adults with hearing loss and whether hearing loss is associated with depression.

The primary objective of our study was to unravel the interrelationships between hearing ability, depressive status (including subclinical and clinical symptoms as potential consequences of hearing loss), cognitive status, and age groups (younger and older adults) in individuals with bilateral sensory onset hearing loss starting in adulthood. Specifically, we investigated whether the severity of depressive symptoms acts as a mediator of cognitive performance. Additionally, we examined potential variations between the younger and older groups regarding secondary depression, cognitive status, and the mediating role of depressive symptoms.

### Primary hypotheses

- There is a correlation between hearing ability (in quiet: hearing threshold dB PTA in the better ear, and in noise: Patient Report Outcome Measure) and
  - Severity (subclinical and clinical) of secondary depressive symptoms.
  - Cognitive performance (episodic memory, executive functioning).
- There is a correlation between severity of depressive symptoms and cognitive performance.
- The severity of secondary depressive symptoms mediates between hearing ability and cognitive performance.

*The following applies to a–c above: At least one connection between an auditory variable, depression and a cognitive variable can be established.*

### Secondary hypotheses

- The main hypothesis applies to both the younger and the older group (Younger 25–54 years, older 55–74 years).
- There are less depressive symptoms in the younger group.
- The cognitive performance is higher in the younger group.

## Materials and methods

### Design and setting

This study is part of a larger cohort study designated to provide information on baseline data before cochlear implantation. It was conducted at Medical University of Hannover, Germany (German Hearing Center) and ENT department of University Hospital Salzburg, Austria, from August 2019 to January 2023. The project was approved by local ethics committees of Hannover (protocol number: 8419_BO_S_2019) and Salzburg (protocol number: 415-E/22489/2-2019).

### Participants and procedure

We recruited cochlear implant^6^ candidates as study participants because they have rather homogeneous levels of hearing loss (with a focus of profound and severe hearing loss). Sixty-one study participants with ages ranging from 25 to 75 years were recruited during outpatient and inpatient care at both centers. All participants with an indication for CI (cochlear implant) surgery in at least one ear according to the Association of the Scientific Medical Societies (AWMF) guidelines for cochlear implantation (77) who provided informed consent for surgery (first CI) were invited to participate in the study if they met the following inclusion criteria: There was symmetrical sensorineural hearing loss in both ears (hearing threshold difference of no more than 20 dB). Hearing loss begins from adulthood onwards (>18 years). The four-frequency PTA (0.5, 1, 2, 4 kHz) in the contralateral ear was at least 40 dB HL. Exclusion criteria comprised unilateral hearing loss, sudden sensorineural hearing loss less than 12 months before CI surgery, blindness, regular use of anticholinergic medication, presence and/or ongoing systemic treatment of malignant disease, an existing diagnosis of primary mood disorder (F30–F39, ICD 10), current psychotic illness (F20–F29) or affective disorder with psychotic symptoms (F 30. 3, F 31.3, F 32.3, F33.3), poor German language skills and very low nonverbal IQ. Primary mood disorders develop before hearing loss and have to be excluded because they may confound cognitive outcomes. As mentioned in the introduction, all mood disorders (primary and secondary as a result of hearing loss) are associated with cognitive decline. Nonverbal IQ was assessed using the Matrizen Test, part of the Wechsler Adult Intelligence Sale—Fourth Edition [WAIS-IV (78, 79)]. If the result of the WMT reached a percentage range of less than 5 percent, the patient was excluded from the study.

**Figure 1:**
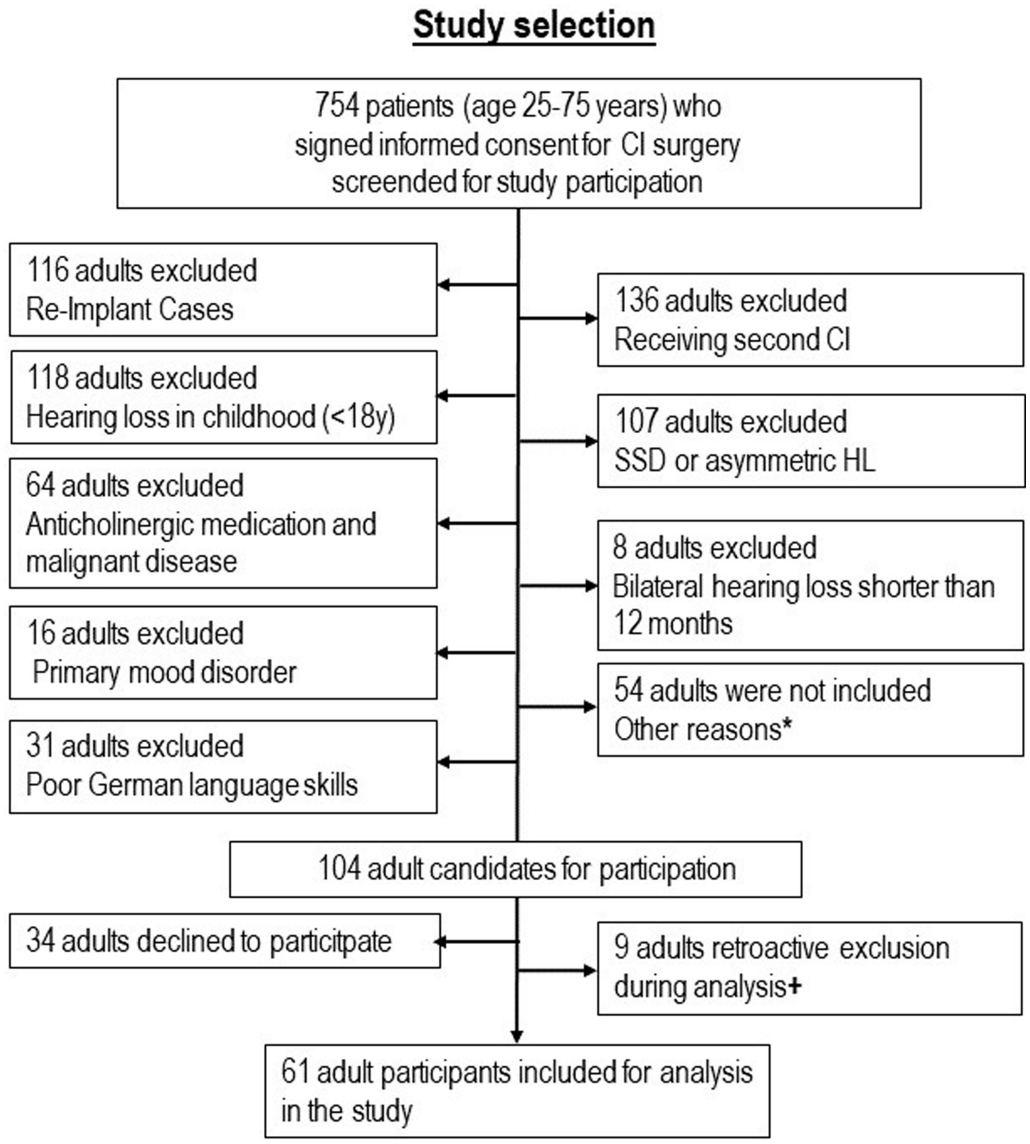
Selection of 61 study participants with hearing loss from adulthood. ^*^Reasons for non-inclusion: 17 because of severe illness and replacement of one investigator, 6 because of illness and vacation of the other investigators, 25 because of logistical and organizational problems in one clinic, 6 for other reasons. + Reasons for retroactive exclusion: 3 because of hearing loss since childhood, 2 because of anticholinergic medication or malignant disease, 1 because of very low nonverbal IQ, 1 because of poor German language skills, 1 because adult was no CI candidate.

The flow chart in Figure 1 shows the selection of participants. Demographic, audiometric, and clinical data of *n* = 61 participants [*n* = 17 younger group (25–54 years), *n* = 44 older group (55–75 years)] are listed in Table 1. Apart from a significant difference in age, there were no differences in demographic variables between the younger group and the older group. All investigations were conducted prior to surgery.

**Table 1:** Demographic, audiometric, educational and clinical data of 61 study participants.

	Older group (*n* = 44)	Younger group (*n* = 17)	Total group (*n* = 61)	*p*
Age (years): mean (SD)	65.18 (6.24)	45.76 (9.47)	59.77 (11.35)	<0.001
Sex	18 male (41%)	8 male (47%)	26 male (43%)	0.78
**Audiometric results (PTA dB HL):**				
Median (min-max)				
Left ear	79.40 (40–110)	78.80 (31–98)	78.80 (31–110)	0.45
Right ear	76.25 (29–120)	70 (56–108)	75 (29–120)	0.37
**Education:**				
Education level^a^, no. (%)				
1	24 (54%)	10 (59%)	34 (56%)	
2	10 (23%)	2 (12%)	12 (20%)	0.34
3	3 (7%)	3 (18%)	6 (10%)	
Educational years, mean (SD)	13.01 (3.10)	14.44 (2.97)	13.41 (3.11)	0.11
**Present risk factor:**				
Nicotine use current, no. (%)	5 (11%)	2 (12%)	7 (11%)	0.99
Nicotine use previous, no. (%)	6 (14%)	4 (24%)	10 (16%)	0.26
Arterial hypertension, no. (%)	17 (39%)	3 (18%)	20 (33%)	0.22
Diabetes mellitus type 1, no. (%)	0	0	0	
Diabetes mellitus type 2, no. (%)	4 (9%)	0	4 (7%)	0.56
Covid infection, no. (%)	0	0		
No Covid infection, no. (%)	8 (18%)	4 (24%)	12 (20%)	
Not requested Covid, no. (%)	36 (82%)	13 (76%)	49 (80%)	
**Cause of hearing loss**^a^, **no. patients**				
Sudden hearing loss	10	2	12	
Otitis media	1	0	1	
Noise	6	0	6	
Acoustic trauma	0	0	0	
Middle ear surgery	3	1	4	
Genetic	1	2	2	
Unknown	15	10	25	
Other	8	2	10	
**Duration of hearing loss**^b^				
Mean (SD), min–max	24.02 (14.04), 1–52	14.35 (7.60), 2–26	21.32 (13.26), 1–52	0.01
**Use of hearing aids**^b^**, no. patients**				
General, no. patients	42	16	58	0.99
Bilateral, no. patients	38	16	54	
No use, no. patients	2	1	3	
**Duration of hearing aid use**^b^**, years, mean (SD), min-max**				
Left ear	13.77 (10.29)	11.73 (8.72)	13.20 (9.85)	0.47
Right ear	14.63 (10.61)	12.02 (8.85)	13.89 (10.14)	0.37
**Speech recognition, Monosyllables with HA in %, mean (SD), min-max**				
Left ear	7.80 (16.05), 0–60	13.33 (22.88), 0–75	9.29 (18.08), 0–75	0.31
Right ear	10.36 (23.82), 0–100	10.71 (17.19), 0–60	10.45 (22.20), 0–100	0.96

SD, standard deviation; PTA, pure tone average (0.5-4 kHz); dB, decibel; HL, hearing level; HA, hearing aid. ^a^1 = secondary school/middle school/junior high, 2 = ordinary level/high school, 3 = university. ^b^Information of the patient.

### Measurements and instruments

The primary outcomes of this study were possible associations between (a) hearing status and severity (subclinical and clinical) of secondary depressive symptoms in younger and older CI candidates, (b) depressive status and cognitive performance, and (c) hearing status and cognitive performance. The secondary outcomes were possible differences in these associations between the younger and older groups.

#### Assessment of hearing status

Hearing assessments were conducted by qualified audiologists in double-walled, soundproof booths, following International Electrotechnical Commission (IEC) regulations. Audiometric tests were performed using equipment that adheres to IEC 60645 standards and is calibrated based on ISO 389 1–9 guidelines. The audiometric tests included measurement of pure tone threshold, with higher thresholds [four-frequency PTA (0.5, 1, 2, 4 kHz) indicating lower hearing status].

Speech recognition was tested with the German Freiburg Numbers (recognition of 10 two-digit recorded numbers) and the Freiburg Monosyllables [recognition of 20 recorded monosyllabic words each per test series (80, 81)]. All speech recognition tests were performed with hearing aids the patients had received from their local acoustician in free-field measurement at 65 dB SPL. Consequently, these patients wore many different HighPower hearing aids. These were not fitted by us nor was any programming changed. The percentage of correctly perceived speech stimuli was documented.

We refrained from a speech recognition test in noise because clinical experience shows that many CI candidates fail a speech test in noise, with and without hearing aids (0% of speech stimuli are correctly recognized), which is due to their severe and profound hearing loss.

As a substitute, we used the “background noise” subscale (BN) of the German version of the APHAB [Abbreviated Profile of Hearing Aid Benefit (82–84)]. This patient-reported outcome measure allows assessment of hearing function in everyday situations. It consists of 24 questions with response options A–G. To analyze the outcomes the answer options A–G are assigned the numbers 1–7. The number 1 stands for pronounced difficulties or restrictions in hearing. The number 7 stands for no difficulties or good subjective hearing. There are four subscales: “Background Noise,” which assesses subjective hearing in noisy situations, “Ease of Communication,” which assesses subjective hearing when communicating in quiet situations, “Reverberation,” which assesses subjective hearing in reverberant situations, and “Aversiveness,” which assesses aversion and discomfort when hearing aversive environmental sounds. Studies show significant correlations between the APHAB total scale, the subscales EC and RV, and the PTA (85–88).

Although we only needed the results of the BN subscale, the participants were asked to answer all 24 questions of the APHAB.

#### Assessment of depressive status

The Beck’s Depression Inventory II (89–91), German version. Kühner et al. (91) consists of four questions on 21 depressive symptoms each. A score of 13 or higher (“clinical severity”) raises suspicion of a mood disorder. We documented the clinical severity and subclinical severity of depressive symptoms (score of 13 and below).

#### Assessment of cognitive status

We tested visual episodic memory and the executive functions working memory, attentional control, inhibition, cognitive flexibility, and phonemic and semantic verbal fluency. We did not examine verbal episodic memory because we could not find a validated test that measures only verbal memory (and not a combination of visual and verbal element), that is suitable for younger and older people, that is also suitable for people with severe and profound hearing loss, and that also has a German version.

We used the short form of the *Non Verbal Learning Test* (*NVLT*), a German test of visual episodic memory based on Kimura’s Recurring Figures Test, which is also available in computer form (92–95). We used the computer form. On the screen, the subject is presented with a series of geometrically shaped or irregularly shaped figures in succession (120 in the short form). For each figure, a decision must be made whether it has been seen before during the test or whether it is being presented for the first time. During the test, 8 of the items shown are repeated a total of five times. Both the number of correct positive answers and the number of false positive answers are evaluated. The difference between correct and false positive answers is documented.

An *n-back* test was used to test attention and working memory. This test goes back to Kirchner et al. (96) and tests (executive functions) control of information flow “active maintenance,” Cohen et al. (97) and the ability to continuously update information. We used the computer version of TAP (98). In this task, participants are presented with numbers on the screen in rapid succession. The task is to determine with a mouse click whether the currently presented number is identical to the previously presented number (*n* = 1), to the previous number (*n* = 2), or even to a previous number (*n* = 3). Number of omissions and the number of errors are documented.

To test focused attention and inhibition the *Go/NoGo* Test was used, developed by Maquire et al. (99). Again, we used the computer version of the TAP (98). In this task, a response “triggered by an external stimulus” must be suppressed in favor “of an internally controlled behavioral response” (Testmanual TAP). In this task, either an x or a + appears on a dark screen. The test person should only press a button when an x is displayed and suppress the impulse to react when a + is displayed. Number of errors and reaction time are documented.

To test cognitive flexibility of executive function, the *Trail Making Test B* (*TMTB*) was used (100–102). In this paper-based test, numbers and letters are to be connected alternately in ascending order as fast as possible. Number of errors and reaction time are documented.

The Regensburg Word Test (103) was used to test phonemic and semantic word fluency [e.g., (104)]. Participants are given the task of producing as many unique words as possible in a given time that begin with a specific letter (e.g., S; phonemic fluency), or belong to one of a specific category (e.g., fruit). The number of words produced correctly was documented.

All cognitive tests were presented visually. Written test instructions were provided for all tests. For communication during test administration (e.g., transitioning from one test to another), there was a template for communication that all investigators had to follow. In Salzburg, the tests were administered by clinical psychologists. In Hannover, the tests were administered by clinical staff who were experienced in communicating with individuals with hearing loss. Since the TAP tests may only be administered by psychologists (see German manual), they were trained by psychologists and then supervised.

#### Assessment of medical variables

To identify potential confounders such as comorbidities or other circumstances and to check the inclusion and exclusion criteria, all participants were examined by medical professionals before the audiological, cognitive and psychological investigations, which also included the general ENT status. Potential exclusion criteria were verified systematically with the help of a self-developed questionnaire, addressing predefined exclusion criteria as mentioned earlier. This questionnaire was filled out by each potential participant before the medical interview and subsequently controlled by medical staff during the examination.

### Changes to the study due to corona pandemic

Since cochlear implantation is not a life-saving procedure, patient volumes at the Hannover and Salzburg clinics declined by more than 50 percent during the COVID-19 pandemic compared to the years before the pandemic. Therefore, the study was extended by 1 year. In Salzburg and in Hannover, the assessments were performed in well-ventilated rooms and partition walls made of Plexiglas between the tester and the participant were used. In Salzburg, the NVLT (Non-verbal Learning Test) was performed on a PC (there was no extra laptop as in Hannover). Since no separation was possible in this test situation, FFP2 masks were worn by the investigators (please note that all test instructions were presented also visually). All participants were required to wear FFP2 masks. As soon as corona testing was available, all patients were tested for corona on admission to the ward.

### Statistics

Statistical analysis was carried out in IBM SPSS Statistics Version 27. Pearson correlations were used in order to assess (i) associations between hearing ability and cognitive functions, (ii) hearing ability and depression, and (iii) depression and cognitive functions. For mediation analyses were planned to use the *mediate* function in the *mediation* package of statistics software R. 4.2.3. In order to assess, whether the correlations addressed in (i)–(iii) differed significantly between the younger and older group, linear multiple regressions were used including age group as a moderator. For cognitive functions, age-adjusted T-values were used as performance estimates. *p*-values were FDR-corrected for multiple comparisons. All missing dada were documented.

## Results

### Association between four frequency hearing thresholds (PTA) and APHAB outcomes

Four-frequency hearing thresholds (PTA) in the better ear were significantly negatively related to subjective hearing ability in noise as assessed with the APHAB (r = −0.31, *p* = 0.02). Participants with objectively worse hearing, i.e., higher PTA, had significantly worse subjective hearing in noise, i.e., lower APHAB scores. Hearing thresholds were furthermore significantly related to subjective hearing in reverberant surroundings (r = −0.30, *p* = 0.02), but not to subjective hearing in quiet [“Ease of communication” (r = 0.08, *p* = 0.56) and also not with subjective hearing of aversive and loud sounds (“Aversiveness”; r = −0.13, *p* = 0.30)].

### Primary outcomes

**Figure 2:**
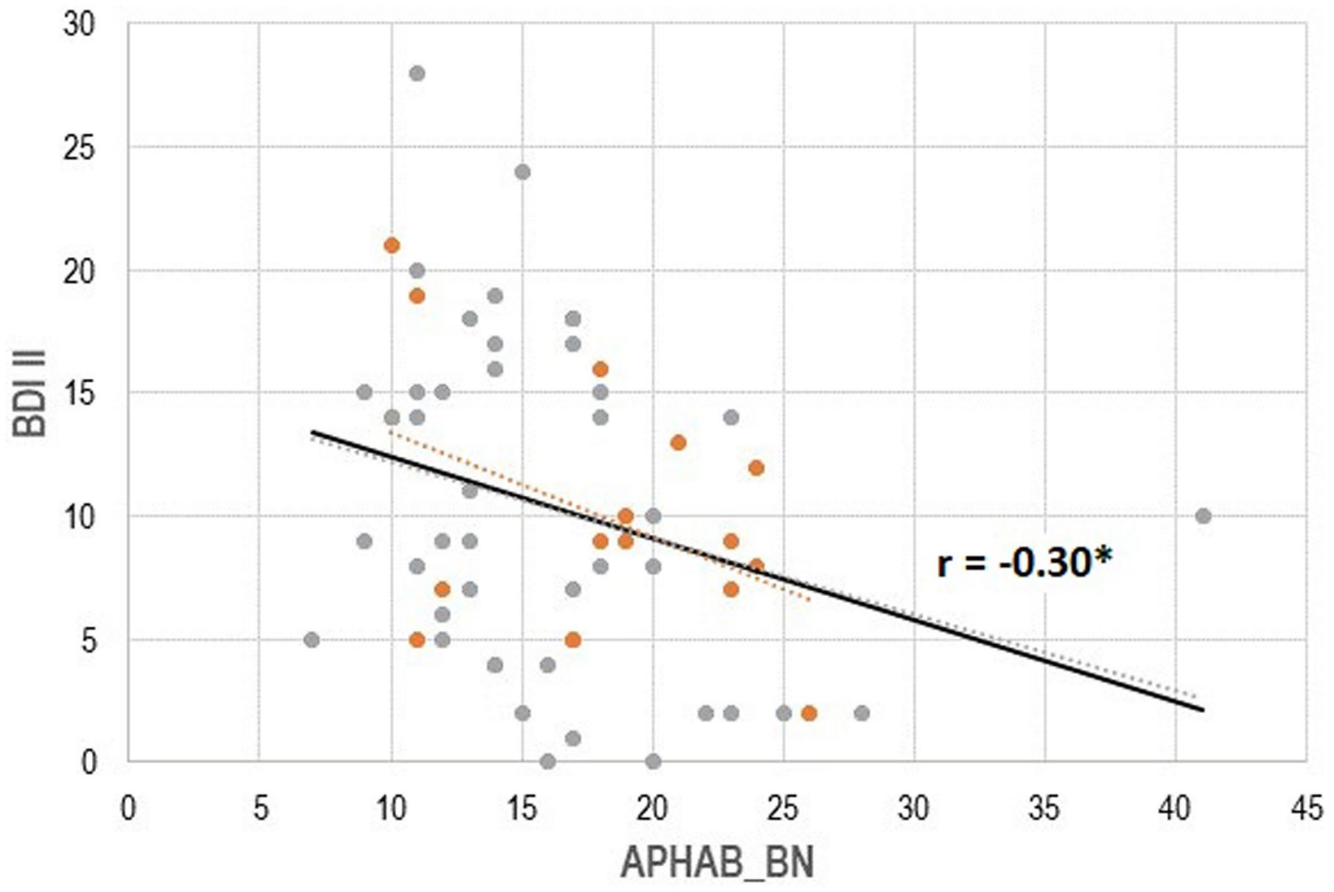
Association between subjective hearing ability in noise (Background Noise, Abbreviated Profile of Hearing Aid Benefit) with severity of depressive symptoms (Becks Depression Inventory II). APHAB: Higher scores indicate better subjective hearing. BDI II: Higher scores indicate more depression. Solid regression line: Total group. *Orange dots*: Younger group (*n* = 17), *gray dots*: Older group (*n* = 44).

As for possible associations between hearing and depression, we found no significant association between four-frequency hearing thresholds (PTA) and BDI II (Beck’s Depression Inventory) results (r = 0.08, 95% CI = [−0.18, 0.33], p_FDR_ = 0.54). Subjective hearing in noise as assessed with the Background Noise subscale (APHAB) was significantly negatively related to depressive symptoms as assessed with the BDI II (r = −0.30, 95% CI [−0.51, −0.052], p_FDR_ = 0.04; see Figure 2): The better the subjective hearing in noise, the less severe are the depressive symptoms.

As for possible associations between hearing and cognitive performance, neither objective hearing ability, as assessed with audiometric testing (PTA, the better ear), nor subjective hearing ability in background noise (BN) were significantly related to any cognitive variables (all │r│< 0.17, all *p* > 0.20).

Furthermore, severity of depressive symptoms was not related to any cognitive variables (Clinical and subclinical cases (*n* = 61), all │r│< 0.23, all *p* > 0.07; clinical cases only, i.e., those that are at or above the cut-off value of 13 (*n* = 21), all │r│ < 0.24, all *p* > 0.29).

Given that no association between depressive symptoms and cognitive variables was observed, and that different aspects of hearing ability related to depressive symptoms and cognitive variables, no mediation analysis was performed.

### Secondary outcomes

The associations between subjective hearing in noise (BN) and severity of depressive symptoms (BDI II) were more pronounced in the younger group (r = −0.44, *p* = 0.07), than in the older group (r = −0.27, *p* = 0.08), though the difference in the associations between groups were not significant (β = 0.34, t = 1.34, *p* = 0.19, compare Figure 2). Associations between BN and cognitive performance did not differ significantly between the older and the younger group (all |β| < 0.57, all |t| < 1.87, all *p* > 0.07; compare Table 2). Associations between the BDI II results and cognitive variables were non-significant in both the younger and older group (all │r│ < 0.38, all *p* > 0.06).

**Table 2:** Comparison between younger group and older group of CI-candidates, Pearson correlations between subjective hearing ability as assessed with the APHAB and cognitive variables, separately by group.

	25–54 (*n* = 17)				55–75 (*n* = 44)			
Task\APHAB	Quiet	Noise	Reverberant	Aversive	Quiet	Noise	Reverberant	Aversive
	EC	BN	RV	AV	EC	BN	RV	AV
RWT phonemic	0.29	0.18	0.20	0.27	0.40^**^	0.07	−0.12	0.28
RWT semantic	0.32	0.00	0.09	0.22	0.25	0.11	0.17	0.42^**^
NVLT	0.75^**^	0.44	0.14	0.23	0.43^**^	−0.08	−0.09	0.16
N-back	0.17	0.01	0.15	0.23	0.27	0.16	0.04	0.28
TMT-B	0.22	−0.26	−0.14	0.46	0.43^**^	0.12	−0.01	0.11
Go/*Nogo t*	0.10	−0.35	−0.06	−0.23	−0.20	0.03	−0.12	−0.37^*^

The results for subjective hearing in noise (BN) are highlighted in green. Asterisks indicate significant results (uncorrected). APHAB, Abbreviated Profile of Hearing Aid Benefit subscales; BN, Background noise; AV, Aversiveness; RV, Reverberant; EC, Ease of Communication; RWT, Regensburger Wortflüssigkeitstest; NVLT, Nonverbal Learning Test; TMTB, Trail Making Test B; t, time.

We also examined possible differences between the younger and older groups in BDI-II scores and cognitive performance. There was no significant difference between the two groups in the severity of depressive symptoms (BDI II, see Table 3). As for cognitive performance, the TMT-B performance was significantly reduced in the older age group compared to the younger age group. However, after correction for multiple comparisons the difference was no longer significant (compare Table 3). No significant difference was found between the younger and older groups on the other cognitive tests (Table 3).

**Table 3:** Cognitive performance in younger adults (*n* = 17) and older adults (*n* = 44) with hearing loss.

	25–54		55–75		All		Comparison			
	Mean	SD	Mean	SD	Mean	SD	T	d	*p*	p_FDR_
BDI II_RS	10.06	5.19	10.37	6.89	10.28	6.41	−0.17	0.05	0.86	0.86
RWT_phonemic PR	31.09	25.48	44.65	23.07	40.87	24.33	−2.00	0.56	0.05	0.14
RWT_semantic PR	37.38	28.86	52.55	27.77	48.32	28.67	−1.89	0.51	0.06	0.14
NVLT_T	49.06	10.39	46.30	10.16	47.07	10.21	0.95	0.27	0.35	0.61
N-back_T	48.82	10.57	50.43	15.24	49.95	13.94	−0.39	0.12	0.70	0.82
TMTB_PR	34.71	31.65	17.27	16.05	22.13	22.67	2.85	0.70	0.01	0.07
Go/NoGo_time_T	48.71	10.32	49.88	10.60	49.55	10.45	−0.39	0.11	0.70	0.82

All were CI-candidates. BDI II, Becks Depression Inventory; RWT, Regensburger Wortflüssigkeitstest; NVLT, Nonverbal Learning Test; TMTB, Trail Making Test B; SD, Standard Deviation; RS, Raw score; PR, Percent range; d, Cohen’s d. *P*-values were FDR-corrected for multiple comparisons (denoted as pFDR).

### Outcomes of unplanned (exploratory) analyses

In exploratory analyses, we looked for possible associations between additional hearing variables, depression (BDI II), and cognitive variables. Additional hearing variables included the remaining three subscales of the APHAB Ease of Communication (EC), Reverberant (RV) and Aversiveness (AV) as well as speech recognition in quiet (Freiburg Monosyllables) and duration of hearing loss (Table 1).

**Figure 3:**
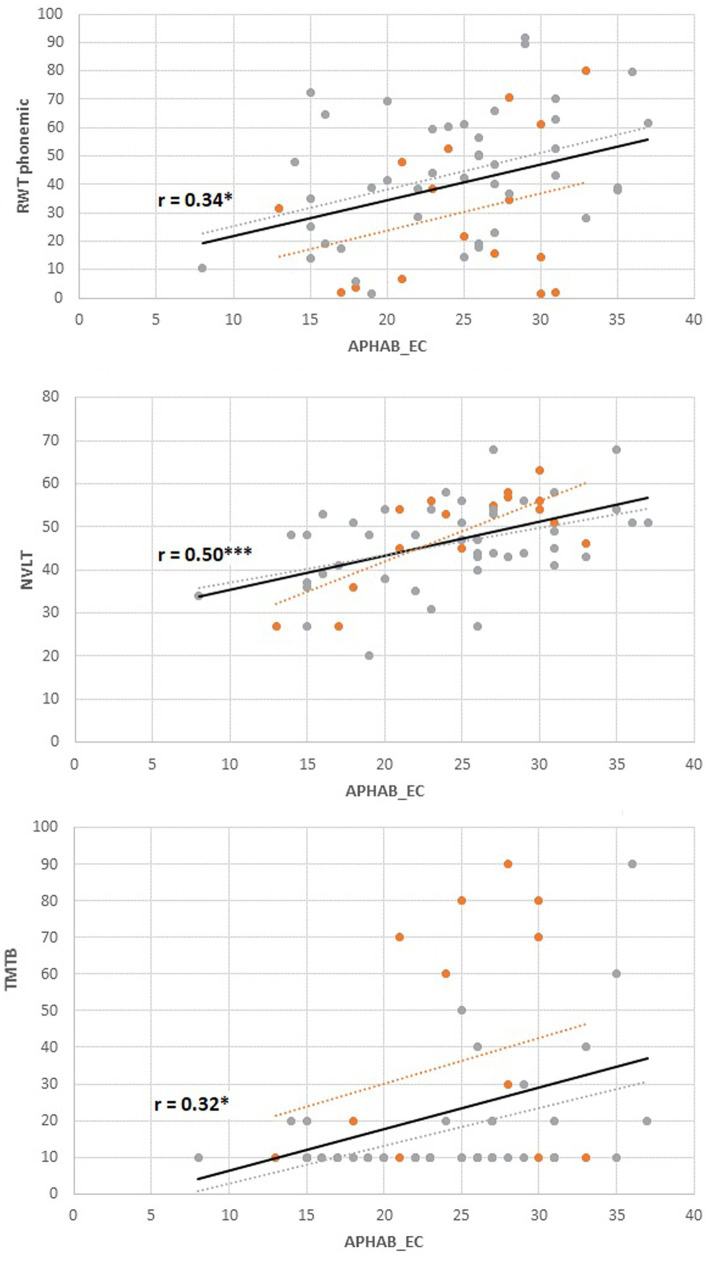
Associations between subjective hearing ability in quiet (Ease of Communication, APHAB) and cognitive performance. *Orange dots*: younger group (*n* = 17), *gray dots*; older group (*n* = 44). Solid regression line: Total group. RWT, Regensburger Wortflüssigkeit, phonemic fluency, percent range; NVLT, Non Verbal Learning Test, T-scores; TMT-B, Trail Making Test B, percent range; APHAB, Higher scores indicate better subjective hearing; Cognitive Tests, Higher scores indicate better results.

As for additional hearing variables and depression, APHAP subjective hearing in quiet (EC), or in reverberant surroundings (RV) were not associated with depressive symptoms (both │r│ < 0.17, both *p* > 0.19), while subjective hearing of aversive and loud sounds (AV) was also significantly negatively related to depressive symptoms (r = −0.31, *p* = 0.02). The duration of hearing loss and speech recognition were not significantly related to depression (duration: r = 0.03, *p* = 0.85; monosyllables: (all │r│ < 0.09, all *p* > 0.48) compare Figure 3).

As for additional hearing variables and cognitive variables, *APHAB subjective hearing in quiet (EC)* was significantly positively associated with several cognitive variables (including phonemic fluency as assessed with the RWT (r = 0.34, p_FDR_ = 0.03), nonverbal visual memory as assessed with the NVLT (r = 0.50, p_FDR_ < 0.001), as well as the TMTB (r = 0.32, p_FDR_ = 0.03), compare Figure 3). Non-significant results were observed for semantic fluency as assessed with the RWT (r = 0.24, p_FDR_ = 0.09), the nback (r = 0.24, p_FDR_ = 0.09) and Go/NoGo-Task (r = −0.14, p_FDR_ = 0.31). *APHAB Subjective hearing of aversive sounds (AV)* was significantly associated with semantic fluency (r = 0.35, p_FDR_ = 0.03) and GoNoGo time (r = −0.34, p_FDR_ = 0.03), but non-significantly to phonemic fluency (r = 0.25, p_FDR_ = 0.07), nonverbal memory (r = 0.17, p_FDR_ = 0.17), n-back performance (r = 0.27, p_FDR_ = 0.07) and TMTB performance (r = 0.22, p_FDR_ = 0.12). *APHAB subjective hearing in reverberant surroundings* (RE) was not significantly related to any cognitive variables (all │r│ < 0.17, all *p* > 0.19). Although some correlations appear numerically larger in the older group, associations did not differ significantly between the older and the younger group (all |β| < 0.45, all |t| < 1.75, all *p* > 0.08; compare Table 2). Duration of hearing loss was not related to any cognitive variable (all │r│< 0.21, all *p* > 0.10). None of the cognitive variables were related to speech test results in quiet (Freiburg Monosyllables; all │r│< 0.23, all *p* > 0.09).

## Discussion

**Figure 4:**
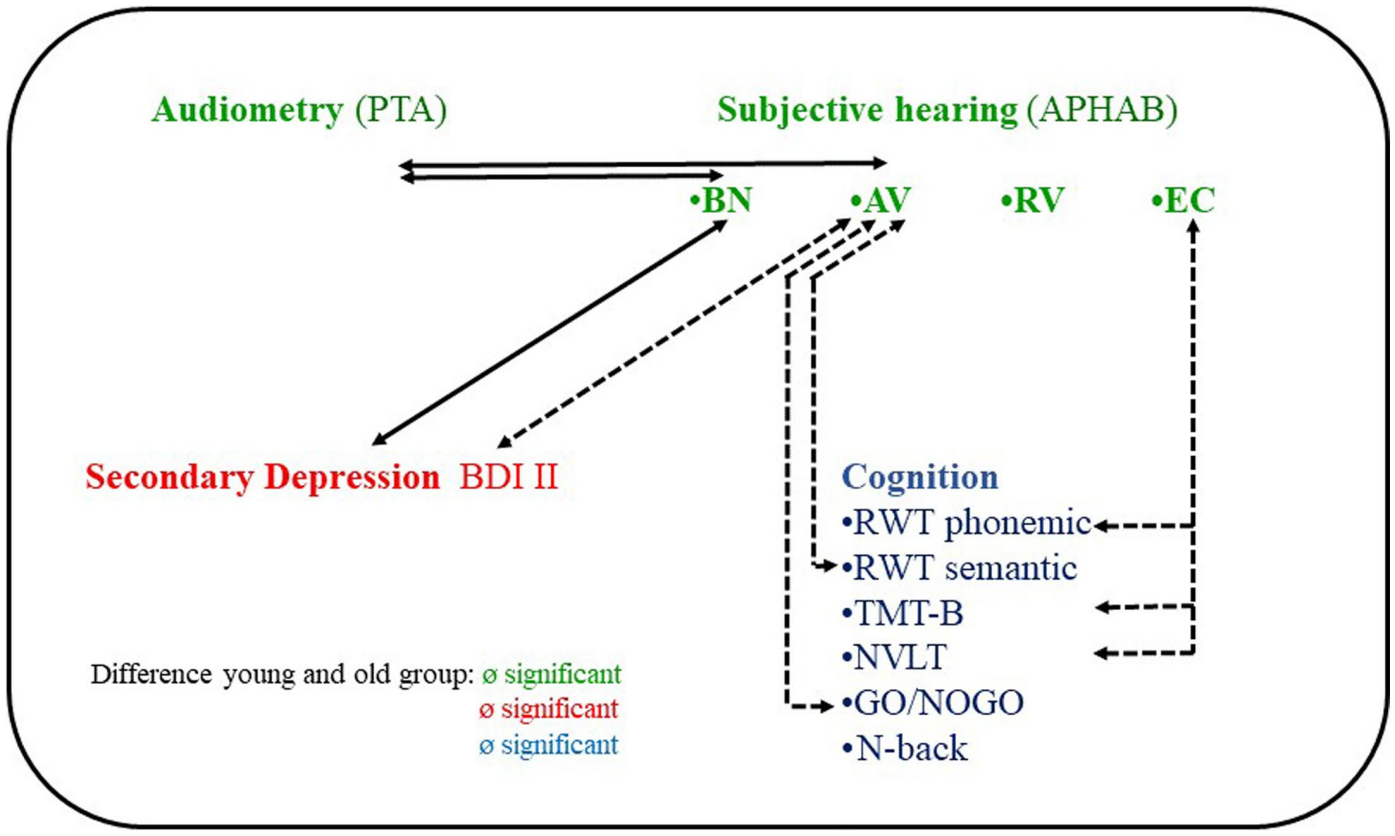
Correlations between hearing, depression and cognitive performance of 61 individuals with hearing loss and cochlear implant candidates. PTA, pure tone average; APHAB, Abbreviated Profile of Hearing Aid Benefit subscales; BN, Background noise; AV, Aversiveness; RV, Reverberant; EC, Ease of Communication; BDI, Becks Depression Inventory; RWT, Regensburger Wortfüssigkeitstest; TMT, Trail Making Test; NVLT, Non Verbal Learning Test.

The present study was designed to assess depressive symptoms as a potential mediator of cognitive impairing effects of hearing loss. In otherwise healthy adults with bilateral hearing loss, all of whom were candidates for cochlear implantation, we found no evidence of a mediating quality of secondary depressive symptoms between hearing loss and cognitive performance. We found a significant association between subjective hearing ability in noise (APHAB) and severity of depressive symptoms, but no significant associations with cognitive performance. Hearing threshold (PTA) was not associated with depression or cognitive performance (Figure 4). There was no association between depressive symptom severity and any cognitive performance. Unexpectedly, there were also no significant differences between the younger and older groups in either depressive symptom severity or cognitive performance.

To our knowledge, the present study was the first to examine the mediator quality of depression in both older people and younger people with hearing loss.

As expected, subjective APHAB hearing in noise was significantly related to the severity of secondary depressive symptoms, result of BDI II.

Speech understanding performance in noise depends on complex central processing and cognitive functions (105–110). Studies on adults without hearing loss suggest that depression may be associated with hearing in noise. For example, Xie et al. found that speech recognition in noise (talker masker) was associated with more errors and higher speech recognition accuracy in young normal hearing adults with major depressive disorder (111). The authors hypothesized that distractibility would be increased in individuals with depression due to speech interference in background noise. These findings were supported by behavioral neuroimaging studies that used both auditory and visual stimuli for distraction (112–115). In all studies, the authors found a higher distractibility in individuals with depressive disorders. Other studies addressed possible associations between speech recognition in noise—audiological test scores—and depressive symptom severity. Carvalho et al. found significant associations between speech perception performance in noise and depressive status in older hearing aid users (116). Heinze-Köhler et al. identified significant associations between short- and long-term speech perception and depressive status in middle-aged and older patients after cochlear implantation (117). Please note that all these studies, including the present study, are correlational studies. Therefore, the question of whether hearing disorders are the cause of depression or whether depression is the cause of hearing disorders cannot be answered.

Unexpectedly, peripheral hearing ability (PTA, the better ear) was not associated with the severity of depressive symptoms. Our results are consistent with those of Gobinath et al. (59) but not consistent with Li et al., Brewster et al., Golub et al., Golub et al., Shukla et al., Chern et al., and Tsimpida et al. (10, 48, 51–57, 118). It should be noted, however, that the nonconsistency refers to the significance of the results and not to the effect sizes. We obtained a small effect size, and this was also the case in these previous studies, with odd ratios ranging from 1.26 (52) to 1.64 (59). These previous studies, with the exception of Gobinath et al. used very large samples (ranging in size from n = 1,204 to 8,529) in which even small effect sizes became significant. With such a large sample, an │r│ of 0.08 as in the present study would most likely have become significant as well.

Furthermore, contrary to our expectations, we did not observe any significant correlation between four frequency hearing thresholds and cognitive performance. Instead, beyond the planned analyses, significant correlations were found between subjective hearing at quiet (APHAB “Ease of communication”) and cognitive performance (Figure 4, for details on the specific cognitive functions, see the discussion of our exploratory analyses below). We attribute this disparity to the fact that the APHAB “Ease of communication” captures different hearing aspects than an audiometric procedure. Hearing thresholds are determined through controlled tests in laboratory-like conditions, evaluating pure tone hearing and hearing with hearing aids without hearing aids (evaluating air conduction, bone conduction, loudness and frequency, best hearing ear)^7^ whereas subjective hearing in everyday situations is determined by a questionnaire. On the other hand, the APHAB subscale “Ease of communication” assesses hearing abilities in real-life verbal communication situations through a questionnaire-based approach, offering a more comprehensive representation of hearing in everyday settings. Hearing in verbal communication situations is assumed to be more cognitively demanding than hearing in audiometric tests of hearing thresholds, we believe it is not only reasonable but also clinically relevant to incorporate hearing variables that closely relate to verbal communication in daily life when investigating potential links between hearing loss and cognitive decline.

Another potentially interesting auditory variable in this context is aversion or misperception when listening to certain environmental sounds (APHAB AV subscale) (82). Beyond the planned analyses, we found significant associations between the APHAB AV and cognitive performance, which is a novel finding (Figure 4). AV differs from the EC and RV subscales in that it is not correlated with audiometric data [hearing thresholds, speech recognition (85–88)]. It also differs from EC, BN, and RV in that it does not correlate with the SSQ [Speech, Spatial, and Qualities of Hearing Scale (84)]. Possible relationships with other hearing variables for example hyperacusis^8^ and noise sensitivity^9^ has not yet been investigated.

We found no significant association between subclinical and clinical severity of depressive symptoms and cognitive performance. The results also do not support the models of Rutherford et al., Uchida et al., and Sharma et al., which hypothesize a direct relationship between depression and cognition of individuals with a hearing loss, see introduction (25, 27, 30). In addition, our findings on secondary depression in people with hearing loss are not consistent with the findings on primary depression in people without hearing loss, see introduction. Depression can be described as a result of genetic predisposition and environmental factors [e.g., (124–126)]. We hypothesize that individuals with secondary depression caused by the critical life event of hearing loss represent a specific group that is distinct from other groups with depression.

There is a well-established consensus that both hearing loss and cognitive decline tend to increase with age. Notably, hearing loss accelerates cognitive decline in older adults, a phenomenon that also has been observed in individuals with normal hearing (14, 21). Given this relationship, one might anticipate that a younger group with hearing loss would outperform an older group with hearing loss on cognitive tests, even after accounting for age differences. However, our study’s findings did not support this hypothesis. To investigate this, we employed age norm scores, such as T scores and percentile ranks, and surprisingly, no significant difference was observed between the younger and older groups with hearing loss. These results challenge the assumption that cognitive decline solely affects older individuals with hearing loss. Instead, our findings lend support to the notion that cognitive decline can also manifest in younger individuals who experience hearing loss. Support for the validity of this hypothesis can be found in a study conducted by Kocabay et al. who compared a small group of younger adults with high-frequency hearing loss (mean age 39 years) with normal-hearing individuals (127). Moreover, it is usually assumed that older individuals are considered to be at increased risk for depression (see the website of US-National Institute on Aging). Unexpectedly, we found no significant differences between young and older groups in depressive status. Our results justify the hypothesis that depression scores are not only elevated in older people with hearing loss (69) but also in younger people.

In addition, there seems to be a widespread assumption that the longer the hearing loss, the more severe the depressive status. In our exploratory analyses, we found no significant association between the years of hearing loss and the severity of depressive symptoms. To our knowledge, there are no other studies on this topic yet. That depressive moods can also occur at any age in people with hearing loss, the use of depression questionnaires such as the BDI are recommended in clinical work.

**Figure 5:**
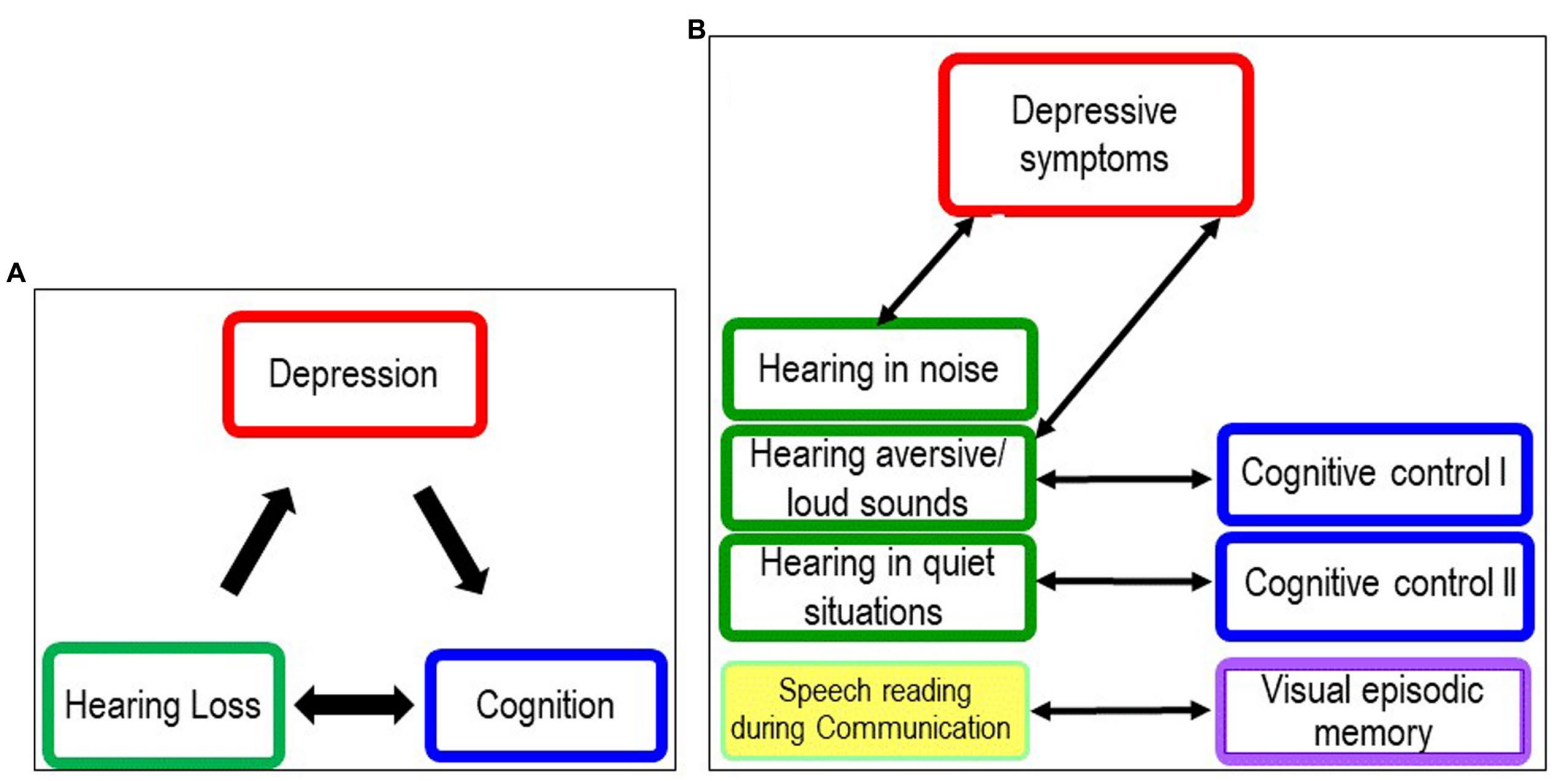
Models illustrating the relationships between hearing loss, depression and cognition. **(A)** Original model Original model at start of study. **(B)** New model following discussion of results of planned analyses and exploratory analyses.

In summary, our hypothesized model that depression mediates between hearing loss and cognition (see Figure 5A) was not supported by our results, neither in the overall group nor in the older or younger groups.

Our results also seem to contrast with a recent study, finding that “depressive symptoms partially mediated the association between hearing loss and cognitive function [standardized regression B coefficient (β) = −0.114; 95% confidence interval (CI): (−0.158, −0.076)] (128). Despite this apparent inconsistency, we believe that there is basically no contradiction between the two studies. The sample of Cao’s studies was large enough (*n* = 8,094) to allow even small effects to become significant.^10^ Our studies would have needed a sample of (*n* = 120) to achieve an effect size of small to medium size and an even larger sample for small effect sizes.^11^ Therefore, the small effects found in Cao et al. (128) could not be detected in our study. Independent from this we have concerns about the validity of the measurement tools used in Cao et al. (128). The presence of hearing loss was only assessed using a single question to study participants (“Do you feel that you have difficulty hearing?”). As mentioned in the introduction, a self-report single question is insufficiently valid for assessing clinical hearing loss (72–75). Furthermore, single question data do not inform about the better and the worse ear or about unilateral deafness. No differentiation can be made between the difference between hearing in quiet and hearing in noise. Moreover, a single test, namely the MMSE (Mini Mental States Examination, Folstein et al.), was used to assess cognitive status (129). Because a substantial part of this test consists of verbal tasks, it is not a sufficiently valid instrument for assessing cognitive performance in individuals with hearing loss (see Introduction).

### Exploratory analyses

Because there were so many unexpected results, exploratory analyses were performed to find possible new paths for further investigation. We found that subjective hearing at quiet (APHAB subscale Ease of Communication) was significantly related to phonemic fluency (Regensburger Wortflüssigkstest), cognitive flexibility (Trail Making Test-B), and visual episodic memory (Non Verbal Learning Test). The better the hearing ability during communication in quiet situations, the higher the cognitive performance.

#### Phonemic fluency

Fluency tests are among the tests of executive functioning or cognitive control. Studies show that these tests require not only controlled processing but also automatic processing (130–132). Previous studies showed that phonemic fluency is significantly associated with the hearing threshold of the better ear (PTA) of younger and older individuals with hearing loss (11) and that older individuals with age related hearing loss seem to have problems especially in the first more automatic part of the task (clustering) (133). The authors suggest that these problems are due to “impoverished auditory input in hearing loss,” which may “disrupt the automatic mapping of acoustic speech to representations in memory, leading to difficulties in implicit recall of such representations” (133).

Following this interpretation, we hypothesize that individuals with hearing loss in adulthood will have problems in automatically retrieving words from the lexicon.^12^ Furthermore, we hypothesize that impoverished auditory input is not only due to hearing loss, but also to social withdrawal and reduced verbal communication as a consequence of hearing loss.

#### Cognitive flexibility

Cognitive flexibility is defined as the ability to change “… perspectives or approaches to a problem, flexibly adjusting to new demands, rules, or priorities (as in switching between tasks)” (134).

Five of the six questions of the APHAB EC subscale refer to verbal interactions. Verbal turn taking requires a very fast switch between the speaker’s perspective and the listener’s perspective, even in a quiet situation. This is complicated by the additional task of planning a response while listening and at the same time anticipating the communication partner’s perspective (135). Additionally the listener “must predict …the end of the current speaker’s turn” to be able to begin with planning (135).

For people with hearing loss in particular this means that cognitive resources are tied up that are lacking elsewhere (136–140). For dialog situations, this likely means that they may be lacking for the cognitive flexibility tasks of perspective taking and task switching. We therefore hypothesize that cognitive load during verbal dialog (also in quiet) impairs the cognitive basis for dialog and thus dialog ability, for example by increasing reaction times when switching between listener and speaker. Furthermore, we expect that people with hearing loss will stop verbal communication when it becomes too demanding and too futile [despite effort, comprehension is no longer possible, see the model of Peele (138)], or avoid communication altogether. Studies in adults without hearing loss have shown that self-perceived stress is negatively related to cognitive control and flexibility (141).

#### Visual episodic memory

We suspect that this correlation with NVLT is not related to hearing (we found no correlation between PTA and NVLT) but to speech reading. In our clinical experience, many patients with severe and profound hearing loss and cochlear implant candidates can read speech because they rely on visual perception (viseme) to follow a conversation. Studies show that a combination of visual and auditory speech information improves speech recognition [without hearing loss (142–144)]. Our hypothesis is that reading speech also trains visual memory. We also interpret the result as a possible indication that the APHAB subscale EC captures not only hearing during communication but also lip-reading ability.

### Strengths and limitations

This appears to be the first time that hearing variables other than traditional hearing thresholds and speech recognition scores have been used in a study addressing cognition in individuals with hearing loss—planned and exploratory. The exploratory part is, in our view, a strength of this study, indicating that it seems reasonable to leave the beaten path of ever-larger study samples and small effects with presumably little clinical relevance. Thus, we turned a supposed disadvantage -our hypotheses could not be confirmed in all respects into an advantage and generated a list of new possible hypotheses (see above).

There are also limitations. This study was conducted during COVID-19 times, which resulted in a decrease of more than 50% in patient volume at the Hannover and Salzburg clinics. As a further consequence, fewer study participants could be recruited than planned, so that the necessary sample size for a mediation analysis was clearly missed (*n* = 120 for a effect seize of an │r│ = 0.25). Extending the study period did not quite produce the expected effect because by the end of the study period, patient volume had not reached pre-Covid levels. A sample size of *n* = 61 may be sufficient to detect moderate to large effects in the associations between hearing and cognition, hearing and depression, and additionally depression and cognition. However, the subgroup of younger adults remained only small. The results of the younger group should therefore only be interpreted with caution.

### Assessment of potential bias

Concerning potential *selection* bias, selection criteria were defined before participant enrollment and were controlled systematically by investigators. All cases of nonparticipation were documented, see flow diagram. However, it cannot be excluded that only the less anxious and less depressed patients entered the clinics during the COVID-19 pandemic. Studies report increased prevalence rates of depression and anxiety during the COVID-19 pandemic (145–147). Concerning potential *performance* bias, a monitor and evaluation plan was created before the investigation. All investigators of the study were monitored by the principal investigator and possible performance errors in the study performance were documented. To control potential bias during cognitive testing, see the procedure described in methods. The influence of the special precautions during the tests (methods) because of Covid is estimated to be low. In the *statistics*, the p’s of all outcomes in the planned analyses were controlled, as well as the p’s of the cognitive outcomes in the exploratory analyses. Note that the exploratory analyses were conducted to find new hypotheses, not to test hypotheses. Additionally, it cannot be excluded that the increased prevalence of depression and anxiety during COVID has minimally influenced the study outcomes of depression. Altogether, the risk of bias in selection, implementation, and statistics can be considered as still low.

### Assessment of external validity

The inclusion and exclusion criteria were quite strict in our study. Our results may not be valid for individuals with hearing loss and a suspected cognitive impairment or with multiple comorbidities. In addition, the results on hearing in noise may be valid only for those who are not remotely able (even with hearing aids) to recognize speech in background noise [four-frequency hearing threshold higher than 35 dB, better ear, best aided (1)]. Additionally cochlear implant candidates may be more engaged than individuals with similar hearing loss who have not considered cochlear implantation.

### Summary and outlook

In summary, the results of the planned and exploratory analyses do not support our original model (see Figure 5A). We found no association between depressive symptoms and hearing loss in adulthood indicated by four-frequency hearing threshold (PTA) and cognitive performance in younger and older cochlear candidates. Instead, we found that subjective hearing limitation in noise was associated with higher depression scores, and subjective limitation in everyday communication was associated with lower cognitive test scores, independent of age (see Figure 5B). As discussed earlier, we hypothesize that the association between hearing in background noise and depression scores is also due to the increased distractibility of depressed patients and the stress caused by distracting information. The association between hearing in quiet and cognitive functions may be attributed to a lack of stimulation from social interactions and isolation when communication skills are affected.

For further studies, it may be useful to consider not only commonly studied auditory variables such as hearing thresholds, but also auditory variables that may play an important role in hearing in everyday life and verbal communication and that have been little studied in research on cognitive decline in individuals with hearing loss to date. This can be done with questionnaires such as the APHAB or other surveys and methods that are part of the ecological momentary assessment (EMA) (148). Another possibility is to study the neural correlates (EEG, MRI) of spoken language or turn-taking in conversations of persons with hearing loss [an example of a study in persons without hearing loss (149)].

### Conclusion

Our study results indicate that depressive symptoms and cognitive impairment are less related to hearing loss *per se*, but more to different aspects of subjective limitations in everyday life due to hearing loss, regardless of age. Examples include distractibility and difficulty concentrating in noisy situations and communication problems in quiet situations that can quickly lead to frustration. These characteristics do not necessarily occur simultaneously in the same person and may affect patients differently depending on their occupation or other life situation.

Reporting of this observational study was done in accordance with the Strengthening the Reporting of Observational Studies in Epidemiology statement (150). A checklist addressing each point may be found as Supplementary material.

## Data availability statement

The raw data supporting the conclusions of this article will be made available by the authors, without undue reservation.

## Ethics statement

The studies involving humans were approved by Hannover (protocol number: 8419_BO_S_2019) and Salzburg (protocol number: 415-E/22489/2-2019). The studies were conducted in accordance with the local legislation and institutional requirements. The participants provided their written informed consent to participate in this study.

## Author contributions

MH: Conceptualization, Funding acquisition, Investigation, Methodology, Project administration, Supervision, Validation, Visualization, Writing – original draft, Writing – review & editing. LR: Investigation, Project administration, Writing – original draft, Writing – review & editing. LW: Conceptualization, Investigation, Methodology, Writing – original draft, Writing – review & editing. BP: Conceptualization, Formal Analysis, Methodology, Software, Validation, Writing – review & editing. SR: Conceptualization, Investigation, Methodology, Writing – original draft, Writing – review & editing. AI: Funding acquisition, Supervision, Visualization, Writing – original draft, Writing – review & editing.

## Footnotes

^1^Better ear: Deal et al., Loughrey et al., Golub et al., Jiang et al., Nicolas et al., and Shende et al. both ears Loughrey et al. and Samelli et al. (11) no information in the remaining studies (5, 7, 10, 13, 14, 20).

^2^As a result of a systematic review and meta-analysis, gray matter and white matter appear to be affected “in almost every region /of the brain” in congenital and acquired hearing loss, with the frontal lobe being most affected (31). In individuals with acquired hearing loss, the insula is most affected (31). Age-Related Hearing Loss (ARHL) belongs to the group of acquired hearing losses. A meta-analysis of older adults with ARHL found that the temporal lobe was predominantly affected (32). Another review found that ARHL is “independently associated with accelerated atrophy of total and regional brain volume” (temporal lobes, particularly in the right hemisphere, entorhinal cortex, anterior cingulate cortex, superior frontal gyrus (associated with working memory), and parts of networks in default mode) and decreased white matter integrity (29). In addition, there are changes in spontaneous neural activity and cerebral functional connectivity of the brain in ARHL (resting-state or task negative networks and task-based or task positive networks). Increased neural activity in the middle frontal gyrus of individuals with presbycusis was found to be correlated with reduced cognitive flexibility (33).

^3^For example, in Rutherford’s model, at the behavioral level, social isolation and loneliness caused by hearing loss in older adults lead to depressive disorder, which in turn is directly related to cognitive impairment. At the neurological level, prefrontal cortex functions are compromised by chronic overuse of the compensatory recruitment of the cognitive control network due to hearing loss. This impairment, combined with reduced or damaged auditory impulses that also damage the primary auditory cortex, negatively impacts the limbic system by affecting emotional responsiveness, emotional control and regulation, and emotional processing. This also leads to depressive disorder.

^4^According to the latest WHO definition, hearing loss starts at a hearing threshold of >20 dB (PTA) https://www.who.int/health-topics/hearing-loss#tab=tab_1.

^5^Cochlear implants (CIs) are electronic medical devices that bypass the hair cells in the cochlea and directly stimulate the auditory nerve (76, 77). They allow people with severe and profound hearing loss “to receive and process sounds and speech” (Medlineplus, National Library of Medicine https://medlineplus.gov/ency/article/007203.htm), where traditional hearing aids have been of little or no use.

^6^“Sound field testing evaluates the composite best hearing ear, which means that the threshold recorded will be the quietest sound audible by either ear at each frequency presented. A normal sound-field audiogram, therefore, does not indicate normal hearing across all tested frequencies in both ears but rather that at each tested frequency, at least one of the ears could hear the tone normally” (78). There are two pathways: Air conduction and bone conduction.

^7^Note that individuals with hearing loss are at a disadvantage in cognitive tasks that are only verbal, e.g., “I’m going to tell you three words to remember. Repeat them after I say all three words.”

^8^“Hyperacusis can be defined as an intolerance to certain everyday sounds that are perceived as too loud or unpleasant and that cause the person significant stress and impairment in his or her daily activities” (119). Hyperacusis is common in patients with tinnitus and also occurs in individuals without hearing loss (120, 121). Although there are several models/theories that address the cause of hyperacusis (121, 122) the phenomenon hyperacusis is not yet fully understood. For example, the central auditory gain model assumes that hyperacusis is the result of an increased (dysfunctional) increase in neural gain that is due to deprived peripheral auditory input. Loudness discomfort level was found to be decreased in individuals with hyperacusis (121).

^9^Noise sensitivity can be described as an “…increased reactivity to sounds that may include general discomfort (annoyance or feeling overwhelmed) due to a perceived noisy environment, regardless of its loudness.” Some authors (123) differed between noise sensitivity and hyperacusis, other authors did not (119, 120).

^10^Studies with very large samples have also often been used to examine possible associations between hearing thresholds (PTA) and cognitive performance (5, 7, 8, 10, 11, 14, 15, 20). Small effect sizes were found in all these studies. Nevertheless, all p’s were significant. [Papers on smaller studies (*n* = 39–119)] looking at the relationship between hearing thresholds and cognition are from Jayakody et al., Nixon et al., Ren et al., and Shende et al. (6, 9, 12, 13).

^11^https://davidakenny.shinyapps.io/MedPower/

^12^A lexicon is the knowledge that a native speaker has about a language” Glossary of Linguistic Terms https://glossary.sil.org/term/lexicon.

## References

[1] Collaborators GHL. Hearing loss prevalence and years lived with disability, 1990-2019: findings from the global burden of disease Study 2019. Lancet. (2021) 397:996–1009. doi: 10.1016/S0140-6736(21)00516-X 33714390 PMC7960691

[2] Blackwell DL Lucas JW Clarke TC. Summary health statistics for U.S. adults: national health interview survey, 2012. Vital Health Stat. (2014) 10:1–161. 24819891

[3] Powell DS Oh ES Reed NS Lin FR Deal JA. Hearing loss and cognition: what we know and where we need to go. Front Aging Neurosci. (2021) 13:769405. doi: 10.3389/fnagi.2021.769405 35295208 PMC8920093

[4] Tay T Wang JJ Kifley A Lindley R Newall P Mitchell P. Sensory and cognitive association in older persons: findings from an older Australian population. Gerontology. (2006) 52:386–94. doi: 10.1159/000095129, PMID: 16921251

[5] Deal JA Betz J Yaffe K Harris T Purchase-Helzner E Satterfield S. Hearing impairment and incident dementia and cognitive decline in older adults: the Health ABC Study. J Gerontol A Biol Sci Med Sci. (2017) 72:703–9. doi: 10.1093/gerona/glw069, PMID: 27071780 PMC5964742

[6] Jayakody DMP Friedland PL Eikelboom RH Martins RN Sohrabi HR. A novel study on association between untreated hearing loss and cognitive functions of older adults: baseline non-verbal cognitive assessment results. Clin Otolaryngol. (2018) 43:182–91. doi: 10.1111/coa.12937, PMID: 28710824

[7] Loughrey DG Kelly ME Kelley GA Brennan S Lawlor BA. Association of age-Related Hearing Loss with Cognitive Function, cognitive impairment, and dementia: a systematic review and meta-analysis. JAMA Otolaryngol Head Neck Surg. (2018) 144:115–26. doi: 10.1001/jamaoto.2017.2513, PMID: 29222544 PMC5824986

[8] Yuan J Sun Y Sang S Pham JH Kong WJ. The risk of cognitive impairment associated with hearing function in older adults: a pooled analysis of data from eleven studies. Sci Rep. (2018) 8:2137. doi: 10.1038/s41598-018-20496-w, PMID: 29391476 PMC5794920

[9] Nixon G Sarant JZ Tomlin D Dowell R. The relationship between peripheral hearing loss and higher order listening function on cognition in older Australians. Int J Audiol. (2019) 58:933–44. doi: 10.1080/14992027.2019.1641752, PMID: 31322017

[10] Golub JS Brickman AM Ciarleglio AJ Schupf N Luchsinger JA. Association of Subclinical Hearing Loss with Cognitive Performance. JAMA Otolaryngol Head Neck Surg. (2020) 146:57–67. doi: 10.1001/jamaoto.2019.3375, PMID: 31725853 PMC6865840

[11] Samelli AG Santos IS Deal JA Brunoni AR Padilha FYOM Matas CG. Hearing loss and cognitive function: baseline findings from the Brazilian longitudinal Study of adult Health: ELSA-brasil. Ear Hear. (2022) 43:1416–25. doi: 10.1097/AUD.0000000000001205, PMID: 35139052

[12] Ren F Luo J Ma W Xin Q Xu L Fan Z. Hearing loss and cognition among older adults in a Han Chinese cohort. Front Neurosci. (2019) 13:632. doi: 10.3389/fnins.2019.00632, PMID: 31293371 PMC6603159

[13] Shende SA Nguyen LT Lydon EA Husain FT Mudar RA. Cognitive flexibility and inhibition in individuals with age-related hearing loss. Geriatrics. (2021) 6:22. doi: 10.3390/geriatrics6010022 33807842 PMC8006052

[14] Nicholas SO Koh EJ Wee SL Eikelboom RH Jayakody DMP Lin F. Peripheral hearing loss and its association with cognition among ethnic Chinese older adults. Dement Geriatr Cogn Disord. (2021) 50:394–400. doi: 10.1159/000519291, PMID: 34592737

[15] Croll PH Vinke EJ Armstrong NM Licher S Vernooij MW Baatenburg de Jong RJ. Hearing loss and cognitive decline in the general population: a prospective cohort study. J Neurol. (2021) 268:860–71. doi: 10.1007/s00415-020-10208-8, PMID: 32910252 PMC7914236

[16] Salthouse TA. Selective review of cognitive aging. J Int Neuropsychol Soc. (2010) 16:754–60. doi: 10.1017/S1355617710000706, PMID: 20673381 PMC3637655

[17] Harada CN Natelson Love MC Triebel KL. Normal cognitive aging. Clin Geriatr Med. (2013) 29:737–52. doi: 10.1016/j.cger.2013.07.002, PMID: 24094294 PMC4015335

[18] Zaninotto P Batty GD Allerhand M Deary IJ. Cognitive function trajectories and their determinants in older people: 8 years of follow-up in the English longitudinal Study of ageing. J Epidemiol Community Health. (2018) 72:685–94. doi: 10.1136/jech-2017-210116, PMID: 29691286 PMC6204948

[19] Waller M Mishra GD Dobson AJ. Estimating the prevalence of dementia using multiple linked administrative health records and capture-recapture methodology. Emerg Themes Epidemiol. (2017) 14:3. doi: 10.1186/s12982-017-0057-3, PMID: 28261312 PMC5327574

[20] Jiang K Armstrong NM Agrawal Y Gross AL Schrack JA Lin FR. Associations of audiometric hearing and speech-in-noise performance with cognitive decline among older adults: the Baltimore longitudinal Study of aging (BLSA). Front Neurol. (2022) 13:1029851. doi: 10.3389/fneur.2022.1029851, PMID: 36570462 PMC9784219

[21] Deal JA Sharrett AR Albert MS Coresh J Mosley TH Knopman D. Hearing impairment and cognitive decline: a pilot study conducted within the atherosclerosis risk in communities neurocognitive study. Am J Epidemiol. (2015) 181:680–90. doi: 10.1093/aje/kwu333, PMID: 25841870 PMC4408947

[22] Li R Miao X Han B Li J. Cortical thickness of the left parahippocampal cortex links central hearing and cognitive performance in aging. Ann N Y Acad Sci. (2023) 1522:117–25. doi: 10.1111/nyas.14971, PMID: 36799333

[23] Lin FR Yaffe K Xia J Xue QL Harris TB Purchase-Helzner E. Hearing loss and cognitive decline in older adults. JAMA Intern Med. (2013) 173:293–9. doi: 10.1001/jamainternmed.2013.1868, PMID: 23337978 PMC3869227

[24] Lin FR Albert M. Hearing loss and dementia—who is listening? Aging Ment Health. (2014) 18:671–3. doi: 10.1080/13607863.2014.915924, PMID: 24875093 PMC4075051

[25] Rutherford BR Brewster K Golub JS Kim AH Roose SP. Sensation and psychiatry: linking age-related hearing loss to late-life depression and cognitive decline. Am J Psychiatry. (2018) 175:215–24. doi: 10.1176/appi.ajp.2017.17040423, PMID: 29202654 PMC5849471

[26] Bowl MR Dawson SJ. Age-related hearing loss. New York, USA: Cold Spring Harbor Perspective Medicine (2019). 9 p. 10.1101/cshperspect.a033217 PMC6671929 30291149

[27] Uchida Y Sugiura S Nishita Y Saji N Sone M Ueda H. Age-related hearing loss and cognitive decline—the potential mechanisms linking the two. Auris Nasus Larynx. (2019) 46:1–9. doi: 10.1016/j.anl.2018.08.010, PMID: 30177417

[28] Slade K Plack CJ Nuttall HE. The effects of age-related hearing loss on the brain and cognitive function. Trends Neurosci. (2020) 43:810–21. doi: 10.1016/j.tins.2020.07.005 32826080

[29] Jafari Z Kolb BE Mohajerani MH. Age-related hearing loss and cognitive decline: MRI and cellular evidence. Ann N Y Acad Sci. (2021) 1500:17–33. doi: 10.1111/nyas.14617, PMID: 34114212

[30] Sharma RK Chern A Golub JS. Age-related hearing loss and the development of cognitive impairment and late-life depression: a scoping overview. Semin Hear. (2021) 42:10–25. doi: 10.1055/s-0041-1725997, PMID: 33883788 PMC8050418

[31] Manno FAM Rodríguez-Cruces R Kumar R Ratnanather JT Lau C. Hearing loss impacts gray and white matter across the lifespan: systematic review, meta-analysis and meta-regression. NeuroImage. (2021) 231:117826. doi: 10.1016/j.neuroimage.2021.117826, PMID: 33549753 PMC8236095

[32] Slade K Reilly JH Jablonska K Smith E Hayes LD Plack CJ. The impact of age-related hearing loss on structural neuroanatomy: a meta-analysis. Front Neurol. (2022) 13:950997. doi: 10.3389/fneur.2022.950997, PMID: 36003293 PMC9393867

[33] Chen YC Chen H Jiang L Bo F Xu JJ Mao CN. Presbycusis disrupts spontaneous activity revealed by resting-state functional MRI. Front Behav Neurosci. (2018) 12:44. doi: 10.3389/fnbeh.2018.00044, PMID: 29593512 PMC5859072

[34] Maharani A Pendleton N Leroi I. Hearing impairment, loneliness, social isolation, and cognitive function: longitudinal analysis using English longitudinal Study on ageing. Am J Geriatr Psychiatry. (2019) 27:1348–56. doi: 10.1016/j.jagp.2019.07.010, PMID: 31402088

[35] McDermott LM Ebmeier KP. A meta-analysis of depression severity and cognitive function. J Affect Disord. (2009) 119:1–8. doi: 10.1016/j.jad.2009.04.022 19428120

[36] Bruder G Kayser J Tenke C. Event-related brain potentials in depression: clinical, cognitive and neurophysiologic implications In: Luck SJ Kappenman ES, editors. Oxford handbook of event-related potential components. New York: Oxford University, Press (2012). 563–92.

[37] Snyder HR. Major depressive disorder is associated with broad impairments on neuropsychological measures of executive function: a meta-analysis and review. Psychol Bull. (2013) 139:81–132. doi: 10.1037/a0028727, PMID: 22642228 PMC3436964

[38] Vu NQ Aizenstein HJ. Depression in the elderly: brain correlates, neuropsychological findings, and role of vascular lesion load. Curr Opin Neurol. (2013) 26:656–61. doi: 10.1097/WCO.0000000000000028 24184971

[39] Roca M Vives M López-Navarro E García-Campayo J Gili M. Cognitive impairments and depression: a critical review. Actas Esp Psiquiatr. (2015) 43:187–93. PMID: 26320897

[40] John A Patel U Rusted J Richards M Gaysina D. Affective problems and decline in cognitive state in older adults: a systematic review and meta-analysis. Psychol Med. (2019) 49:353–65. doi: 10.1017/S0033291718001137, PMID: 29792244 PMC6331688

[41] Zainal NH Newman MG. Elevated anxious and depressed mood relates to future executive dysfunction in older adults: a longitudinal network analysis of psychopathology and cognitive functioning. Clin Psychol Sci. (2023) 11:218–38. doi: 10.1177/21677026221114076, PMID: 36993876 PMC10046395

[42] Koenig AM Bhalla RK Butters MA. Cognitive functioning and late-life depression. J Int Neuropsychol Soc. (2014) 20:461–7. doi: 10.1017/S1355617714000198, PMID: 24685173 PMC4107679

[43] Lai S Zhong S Wang Y Zhang Y Xue Y Zhao H. The prevalence and characteristics of MCCB cognitive impairment in unmedicated patients with bipolar II depression and major depressive disorder. J Affect Disord. (2022) 310:369–76. doi: 10.1016/j.jad.2022.04.153, PMID: 35504401

[44] Vollmert C Tost H Brassen S Jatzko A Braus DF. Depression and modern neuroimaging. Fortschr Neurol Psychiatr. (2004) 72:435–45. doi: 10.1055/s-2004-818398, PMID: 15305238

[45] Zhao H Lai S Zhong S Zhang Y Yang H Jia Y. Variation in thyroid-stimulating hormone and cognitive disorders in unmedicated middle-aged patients with major depressive disorder: a proton magnetic resonance spectroscopy Study. Mediat Inflamm. (2022) 2022:1–12. doi: 10.1155/2022/1623478 PMC9467792 36105682

[46] Lu X Lai S Luo A Huang X Wang Y Zhang Y. Biochemical metabolism in the anterior cingulate cortex and cognitive function in major depressive disorder with or without insomnia syndrome. J Affect Disord. (2023) 335:256–63. doi: 10.1016/j.jad.2023.04.132 37164065

[47] Wulff L. Amygdala-Volumenveränderung bei Depression? Entwicklung und Anwendung eines Segmentierprotokolls für hochauflösende. Marburg, Germany: MRT Philipps-Universität Marburg (2018).

[48] Li CM Zhang X Hoffman HJ Cotch MF Themann CL Wilson MR. Hearing impairment associated with depression in US adults, National Health and nutrition examination survey 2005-2010. JAMA Otolaryngol Head Neck Surg. (2014) 140:293–302. doi: 10.1001/jamaoto.2014.42, PMID: 24604103 PMC4102382

[49] Kim SY Kim HJ Park EK Joe J Sim S Choi HG. Severe hearing impairment and risk of depression: a national cohort study. PLoS One. (2017) 12:e0179973. doi: 10.1371/journal.pone.0179973, PMID: 28640916 PMC5481021

[50] Kim HJ Jeong S Roh KJ Oh YH Suh MJ. Association between hearing impairment and incident depression: a Nationwide follow-up Study. Laryngoscope. (2023). doi: 10.1002/lary.30654, PMID: 36896880

[51] Brewster KK Ciarleglio A Brown PJ Chen C Kim HO Roose SP. Age-related hearing loss and its association with depression in later life. Am J Geriatr Psychiatry. (2018) 26:788–96. doi: 10.1016/j.jagp.2018.04.003, PMID: 29752060 PMC6008216

[52] Brewster KK Hu MC Zilcha-Mano S Stein A Brown PJ Wall MM. Age-related hearing loss, late-life depression, and risk for incident dementia in older adults. J Gerontol A Biol Sci Med Sci. (2021) 76:827–34. doi: 10.1093/gerona/glaa242, PMID: 32959064 PMC8427720

[53] Golub JS Brewster KK Brickman AM Ciarleglio AJ Kim AH Luchsinger JA. Association of Audiometric age-Related Hearing Loss with Depressive Symptoms among Hispanic Individuals. JAMA Otolaryngol Head Neck Surg. (2019) 145:132–9. doi: 10.1001/jamaoto.2018.3270, PMID: 30520955 PMC6396846

[54] Golub JS Brewster KK Brickman AM Ciarleglio AJ Kim AH Luchsinger JA. Subclinical hearing loss is associated with depressive symptoms. Am J Geriatr Psychiatry. (2020) 28:545–56. doi: 10.1016/j.jagp.2019.12.008, PMID: 31980375 PMC7324246

[55] Shukla A Reed NS Armstrong NM Lin FR Deal JA Goman AM. Hearing loss, hearing aid use, and depressive symptoms in older adults-findings from the atherosclerosis risk in communities neurocognitive Study (ARIC-NCS). J Gerontol B Psychol Sci Soc Sci. (2021) 76:518–23. doi: 10.1093/geronb/gbz128, PMID: 31628485 PMC7887727

[56] Chern A Irace AL Golub JS. The laterality of age-related hearing loss and depression. Otol Neurotol. (2022) 43:625–31. doi: 10.1097/MAO.0000000000003531, PMID: 35709424 PMC9467465

[57] Tsimpida D Kontopantelis E Ashcroft DM Panagioti M. The dynamic relationship between hearing loss, quality of life, socioeconomic position and depression and the impact of hearing aids: answers from the English longitudinal Study of ageing (ELSA). Soc Psychiatry Psychiatr Epidemiol. (2022) 57:353–62. doi: 10.1007/s00127-021-02155-0, PMID: 34383085 PMC8784360

[58] Tseng CC Hu LY Liu ME Yang AC Shen CC Tsai SJ. Risk of depressive disorders following sudden sensorineural hearing loss: a nationwide population-based retrospective cohort study. J Affect Disord. (2016) 197:94–9. doi: 10.1016/j.jad.2016.03.020 26985740

[59] Gopinath B Wang JJ Schneider J Burlutsky G Snowdon J McMahon CM. Depressive symptoms in older adults with hearing impairments: the Blue Mountains Study. J Am Geriatr Soc. (2009) 57:1306–8. doi: 10.1111/j.1532-5415.2009.02317.x, PMID: 19570163

[60] Andresen EM Malmgren JA Carter WB Patrick DL. Screening for depression in well older adults: evaluation of a short form of the CES-D (Center for Epidemiologic Studies Depression Scale). Am J Prev Med. (1994) 10:77–84. doi: 10.1016/S0749-3797(18)30622-6, PMID: 8037935

[61] Tang TY Luan Y Jiao Y Zhang J Ju SH Teng GJ. Disrupted amygdala connectivity is associated with elevated anxiety in sensorineural hearing loss. Front Neurosci. (2020) 14:616348. doi: 10.3389/fnins.2020.616348, PMID: 33362462 PMC7758419

[62] Aoki M Okuda H Ishihara H Hayashi H Ohashi T Nishihori T. Hearing loss is associated with hippocampal atrophy and high cortisol/dehydroepiandrosterone sulphate ratio in older adults. Int J Audiol. (2021) 60:293–9. doi: 10.1080/14992027.2020.1831703, PMID: 33100039

[63] Brewster K Choi CJ He X Kim AH Golub JS Brown PJ. Hearing rehabilitative treatment for older adults with comorbid hearing loss and depression: effects on depressive symptoms and executive function. Am J Geriatr Psychiatry. (2022) 30:448–58. doi: 10.1016/j.jagp.2021.08.006, PMID: 34489159 PMC8841567

[64] Falkai P Wittchen HU. Diagnostisches und statistisches Manual psychischer Störungen DSM-5. Göttingen: Hogrefe (2015).

[65] Steinberg LJ Underwood MD Bakalian MJ Kassir SA Mann JJ Arango V. 5-HT1A receptor, 5-HT2A receptor and serotonin transporter binding in the human auditory cortex in depression. J Psychiatry Neurosci. (2019) 44:294–302. doi: 10.1503/jpn.180190, PMID: 31120232 PMC6710086

[66] Kang HH Wang CH Chen HC Li IH Cheng CY Liu RS. Investigating the effects of noise-induced hearing loss on serotonin transporters in rat brain using 4-[18F]-ADAM/small animal PET. NeuroImage. (2013) 75:262–9. doi: 10.1016/j.neuroimage.2012.06.049, PMID: 22766166

[67] Li IH Shih JH Jhao YT Chen HC Chiu CH Chen CF. Regulation of noise-induced loss of serotonin transporters with resveratrol in a rat model using 4-[18F]-ADAM/small-animal positron emission tomography. Molecules. (2019) 24:1344. doi: 10.3390/molecules24071344 30959762 PMC6480549

[68] Keesom SM Hurley LM. Silence, solitude, and serotonin: neural mechanisms linking hearing loss and social isolation. Brain Sci. (2020) 10:367. doi: 10.3390/brainsci10060367, PMID: 32545607 PMC7349698

[69] Huber M Roesch S Pletzer B Lukaschyk J Lesinski-Schiedat A Illg A. Cognition in older adults with severe to profound sensorineural hearing loss compared to peers with normal hearing for age. Int J Audiol. (2020) 59:254–62. doi: 10.1080/14992027.2019.1687947, PMID: 31718333

[70] Powell DS Brenowitz WD Yaffe K Armstrong NM Reed NS Lin FR. Examining the combined estimated effects of hearing loss and depressive symptoms on risk of cognitive decline and incident dementia. J Gerontol B Psychol Sci Soc Sci. (2022) 77:839–49. doi: 10.1093/geronb/gbab194, PMID: 34655295 PMC9071460

[71] Disoriders NIoDaOC. What the numbers mean: An epidemiological perspective on hearing. (2011). Available at: www.nidcd.nih.gov/health/statistics/what-numbers-mean-epidemiological-perspective-hearing#measuring.

[72] Kirk KM McGuire A Nasveld PE Treloar SA. Comparison of self-reported and audiometrically-measured hearing loss in the Australian defence force. Int J Audiol. (2012) 51:294–8. doi: 10.3109/14992027.2011.625981, PMID: 22149463

[73] Choi JE Moon IJ Baek SY Kim SW Cho YS. Discrepancies between self-reported hearing difficulty and hearing loss diagnosed by audiometry: prevalence and associated factors in a national survey. BMJ Open. (2019) 9:e022440. doi: 10.1136/bmjopen-2018-022440, PMID: 31048419 PMC6501946

[74] Curti SA Taylor EN Su D Spankovich C. Prevalence of and characteristics associated with self-reported good hearing in a population with elevated audiometric thresholds. JAMA Otolaryngol Head Neck Surg. (2019) 145:626–33. doi: 10.1001/jamaoto.2019.1020, PMID: 31169892 PMC6555479

[75] Heigl K Gerstner D Huß J Weilnhammer V Jenkac C Perez-Alvarez C. The validity of using a self-report single question as a means to detect hearing loss in an adolescent population. Int J Audiol. (2022) 1-8:1–8. doi: 10.1080/14992027.2022.2129852 36271818

[76] Armstrong NM An Y Doshi J Erus G Ferrucci L Davatzikos C. Association of Midlife Hearing Impairment with Late-Life Temporal Lobe Volume Loss. JAMA Otolaryngol Head Neck Surg. (2019) 145:794–802. doi: 10.1001/jamaoto.2019.1610, PMID: 31268512 PMC6613307

[77] Deutsche Gesellschaft für Hals-Nasen-Ohren-Heilkunde, Kopf-und Hals-Chirurgie eV. AWMF S2k-Leitlinie Cochlea-Implantat Versorgung Bonn2020. (2020) Available at: www.awmf.org/.

[78] Wechsler D. Wechsler Adult Intelligence Scale. 4th ed. Frankfurt: Petermann, F. (2012).

[79] Petermann F Lepach AC. Wechsler Memory Scale – Fourth Edition(WMS-IV) Manual zur Durchführung und Auswertung. F rankfurt: Pearson Assessment & Information GmbH (2012).

[80] Hesse G Janssen T Kinkel M Mrowinski D Müller-Deile J Ptok M. Praxis der Audiometrie. Stuttgart: Georg Thieme Verlag (2009).

[81] Lehnhardt EL. Praxis Der Audiometrie. Stuttgart: Georg Thieme Verlag (2001).

[82] Cox RM Alexander GC. The abbreviated profile of hearing aid benefit. Ear Hear. (1995) 16:176–86. doi: 10.1097/00003446-199504000-00005 7789669

[83] Löhler J Moser L Heinrich D Hörmann K Walther LE. Results of clinical use of the German version of the APHAB. HNO. (2012) 60:626–36. doi: 10.1007/s00106-011-2466-x, PMID: 22763767

[84] Srinivasan N O'Neill S. Comparison of speech, spatial, and qualities of hearing scale (SSQ) and the abbreviated profile of hearing aid benefit (APHAB) questionnaires in a large cohort of self-reported Normal-hearing adult listeners. Audiol Res. (2023) 13:143–50. doi: 10.3390/audiolres13010014, PMID: 36825952 PMC9952610

[85] Löhler J Akcicek B Wollenberg B Kappe T Schlattmann P Schönweiler R. The influence of frequency-dependent hearing loss to unaided APHAB scores. Eur Arch Otorhinolaryngol. (2016) 273:3587–93. doi: 10.1007/s00405-016-3966-9, PMID: 26975446

[86] Brännström KJ Andersson K Sandgren O Whitling S. Clinical application and psychometric properties of a Swedish translation of the abbreviated profile of hearing aid benefit. J Am Acad Audiol. (2020) 31:656–65. doi: 10.1055/s-0040-1718702, PMID: 33296928

[87] Dornhoffer JR Meyer TA Dubno JR McRackan TR. Assessment of hearing aid benefit using patient-reported outcomes and audiologic measures. Audiol Neurootol. (2020) 25:215–23. doi: 10.1159/000506666, PMID: 32241007 PMC7371552

[88] Cox RM Alexander GC Gray GA. Audiometric correlates of the unaided APHAB. J Am Acad Audiol. (2003) 14:361–71. doi: 10.1055/s-0040-1715755, PMID: 14620610

[89] Wang YP Gorenstein C. Psychometric properties of the Beck depression inventory-II: a comprehensive review. Braz J Psychiatry. (2013) 35:416–31. doi: 10.1590/1516-4446-2012-1048, PMID: 24402217

[90] At B Ch W Mendelson M Mock J Erbaugh J. An inventory for measuring depression. Arch Gen Psychiatry. (1961) 4:561–71. doi: 10.1001/archpsyc.1961.01710120031004 13688369

[91] Kühner C Bürger C Keller F Hautzinger M. Reliability and validity of the revised Beck depression inventory (BDI-II). Results from German samples. Nervenarzt. (2007) 78:651–6. doi: 10.1007/s00115-006-2098-7, PMID: 16832698

[92] Sturm W Willlmes K. WienerTestsystem. Nonverbaler Lerntest (NVLT). Mödling: Schuhfried GmbH (2018).

[93] Kindsvater S Sturm W. Computer- vs papier-Bleistiftvorgabe: Äquivalenzstudie zum nonverbalen Lerntest (NVLT) [computerised vs paper-pencil testing: a study concerning the equivalence of these two test procedures for the nonverbal learning test (NVLT)]. Z Neuropsychol. (2003) 14:13–21. doi: 10.1024//1016-264X.14.1.13

[94] Sturm W Willmes K. Nonverbaler Lerntest (NVLT). Göttingen: Hogrefe (1999).

[95] Kimura D. Right temporal-lobe damage. Perception of unfamiliar stimuli after damage. Arch Neurol. (1963) 8:264–71. doi: 10.1001/archneur.1963.00460030048004 14032748

[96] Wk K. Age differences in short-term retention of rapidly changing information. J Exp Psychol. (1958) 55:352–8. doi: 10.1037/h0043688, PMID: 13539317

[97] Cohen JD Perlstein WM Braver TS Nystrom LE Noll DC Jonides J. Temporal dynamics of brain activation during a working memory task. Nature. (1997) 386:604–8. doi: 10.1038/386604a0 9121583

[98] Zimmermann P Fimm B. Testbatterie zur Aufmerksamkeitsprüfung (TAP) Version 2.3.1. Herzogenrath, Germany: Psytest (2019).

[99] Maguire MJ Brier MR Moore PS Ferree TC Ray D Mostofsky S. The influence of perceptual and semantic categorization on inhibitory processing as measured by the N2-P3 response. Brain Cogn. (2009) 71:196–203. doi: 10.1016/j.bandc.2009.08.018, PMID: 19773108 PMC2783209

[100] Giovagnoli AR Del Pesce M Mascheroni S Simoncelli M Laiacona M Capitani E. Trail making test: normative values from 287 normal adult controls. Ital J Neurol Sci. (1996) 17:305–9. doi: 10.1007/BF01997792, PMID: 8915764

[101] Tombaugh TN. Trail making test a and B: normative data stratified by age and education. Arch Clin Neuropsychol. (2004) 19:203–14. doi: 10.1016/S0887-6177(03)00039-8, PMID: 15010086

[102] Reitan RM. Validity of the trail making test as an indicator of organic brain damage. Percept Mot Skills. (1958) 8:271–6. doi: 10.2466/pms.1958.8.3.271

[103] Aschenbrenner S Oliver T Klaus L. Regensburger Wortflüssigkeits-Test (RWT). Göttingen, Bern, Toronto, Seattle: Hogrefe Verlag für Psychologie (2000).

[104] Baldo JV Schwartz S Wilkins D Dronkers NF. Role of frontal versus temporal cortex in verbal fluency as revealed by voxel-based lesion symptom mapping. J Int Neuropsychol Soc. (2006) 12:896–900. doi: 10.1017/S1355617706061078, PMID: 17064451

[105] Pichora-Fuller MK Schneider BA Daneman M. How young and old adults listen to and remember speech in noise. J Acoust Soc Am. (1995) 97:593–608. doi: 10.1121/1.412282, PMID: 7860836

[106] Song JH Skoe E Banai K Kraus N. Perception of speech in noise: neural correlates. J Cogn Neurosci. (2011) 23:2268–79. doi: 10.1162/jocn.2010.21556, PMID: 20681749 PMC3253852

[107] Souza P Arehart K Miller CW Muralimanohar RK. Effects of age on F0 discrimination and intonation perception in simulated electric and electroacoustic hearing. Ear Hear. (2011) 32:75–83. doi: 10.1097/AUD.0b013e3181eccfe9, PMID: 20739892 PMC3010262

[108] Shojaei E Ashayeri H Jafari Z Zarrin Dast MR Kamali K. Effect of signal to noise ratio on the speech perception ability of older adults. Med J Islam Repub Iran. (2016) 30:342. PMID: 27390712 PMC4898833

[109] Wong PC Ettlinger M Sheppard JP Gunasekera GM Dhar S. Neuroanatomical characteristics and speech perception in noise in older adults. Ear Hear. (2010) 31:471–9. doi: 10.1097/AUD.0b013e3181d709c2 20588117 PMC2919052

[110] Halevi-Katz DN Yaakobi E Putter-Katz H. Exposure to music and noise-induced hearing loss (NIHL) among professional pop/rock/jazz musicians. Noise Health. (2015) 17:158–64. doi: 10.4103/1463-1741.155848, PMID: 25913555 PMC4918652

[111] Xie Z Zinszer BD Riggs M Beevers CG Chandrasekaran B. Impact of depression on speech perception in noise. PLoS One. (2019) 14:e0220928. doi: 10.1371/journal.pone.0220928, PMID: 31415624 PMC6695097

[112] Cornblatt BA Lenzenweger MF Erlenmeyer-Kimling L. The continuous performance test, identical pairs version: II. Contrasting attentional profiles in schizophrenic and depressed patients. Psychiatry Res. (1989) 29:65–85. doi: 10.1016/0165-1781(89)90188-1, PMID: 2772099

[113] Lemelin S Baruch P Vincent A Everett J Vincent P. Distractibility and processing resource deficit in major depression. Evidence for two deficient attentional processing models. J Nerv Ment Dis. (1997) 185:542–8. doi: 10.1097/00005053-199709000-00002, PMID: 9307615

[114] Lepistö T Soininen M Ceponiene R Almqvist F Näätänen R Aronen ET. Auditory event-related potential indices of increased distractibility in children with major depression. Clin Neurophysiol. (2004) 115:620–7. doi: 10.1016/j.clinph.2003.10.020 15036058

[115] Desseilles M Balteau E Sterpenich V Dang-Vu TT Darsaud A Vandewalle G. Abnormal neural filtering of irrelevant visual information in depression. J Neurosci. (2009) 29:1395–403. doi: 10.1523/JNEUROSCI.3341-08.2009, PMID: 19193886 PMC6666064

[116] De Carvalho LM Gonsalez EC Iorio MC. Speech perception in noise in the elderly: interactions between cognitive performance, depressive symptoms, and education. Braz J Otorhinolaryngol. (2017) 83:195–200. doi: 10.1016/j.bjorl.2016.03.017, PMID: 27177979 PMC9442690

[117] Heinze-Köhler K Lehmann EK Hoppe U. Depressive symptoms affect short- and long-term speech recognition outcome in cochlear implant users. Eur Arch Otorhinolaryngol. (2021) 278:345–51. doi: 10.1007/s00405-020-06096-3, PMID: 32504200 PMC7826306

[118] Brewster KK Rutherford BR. Hearing loss, psychiatric symptoms, and cognitive decline: An increasingly important triad in older adults. Am J Geriatr Psychiatry. (2021) 29:554–6. doi: 10.1016/j.jagp.2020.10.015 33153873 PMC8081731

[119] Kula FB Cropley M Aazh H. Hyperacusis and Misophonia: a systematic review of psychometric measures. J Am Acad Audiol. (2023) 33. doi: 10.1055/a-1896-5032 35817311

[120] Aazh H Moore BC Lammaing K Cropley M. Tinnitus and hyperacusis therapy in a UK National Health Service audiology department: Patients' evaluations of the effectiveness of treatments. Int J Audiol. (2016) 55:514–22. doi: 10.1080/14992027.2016.1178400, PMID: 27195947 PMC4950421

[121] Coey JG De Jesus O. Hyperacusis. Treasure Island, Florida, USA: StatPearls (2023).

[122] Knipper M Van Dijk P Nunes I Rüttiger L Zimmermann U. Advances in the neurobiology of hearing disorders: recent developments regarding the basis of tinnitus and hyperacusis. Prog Neurobiol. (2013) 111:17–33. doi: 10.1016/j.pneurobio.2013.08.002, PMID: 24012803

[123] Henry JA Theodoroff SM Edmonds C Martinez I Myers PJ Zaugg TL. Sound tolerance conditions (hyperacusis, Misophonia, noise sensitivity, and phonophobia): definitions and clinical management. Am J Audiol. (2022) 31:513–27. doi: 10.1044/2022_AJA-22-00035 35858241

[124] Gronewold J Duman EE Engel M Engels M Siegrist J Erbel R. Association between life events and later depression in the population-based Heinz Nixdorf recall study-the role of sex and optimism. PLoS One. (2022) 17:e0271716. doi: 10.1371/journal.pone.0271716, PMID: 35857802 PMC9299341

[125] School HM. How genes and life events affect mood and depression: Harvard Health Publishing; (2022) Available at: https://www.health.harvard.edu/depression/how-genes-and-life-events-affect-mood-and-depression.

[126] Coleman JRI Gaspar HA Bryois J Breen G Consortium BDWGotPG, Consortium MDDWGotPG. The genetics of the mood disorder Spectrum: genome-wide association analyses of more than 185,000 cases and 439,000 controls. Biol Psychiatry. (2020) 88:169–84. doi: 10.1016/j.biopsych.2019.10.015 31926635 PMC8136147

[127] Kocabay AP Aslan F Yüce D Turkyilmaz D. Speech in noise: implications of age, hearing loss, and cognition. Folia Phoniatr Logop. (2022) 74:345–51. doi: 10.1159/000525580 35738235

[128] Cao X Liu Q Liu J Yang B Zhou J. The impact of hearing loss on cognitive impairment: the mediating role of depressive symptoms and the moderating role of social relationships. Front Public Health. (2023) 11:1149769. doi: 10.3389/fpubh.2023.1149769, PMID: 37089498 PMC10116415

[129] Folstein MF Folstein SE McHugh PR. "mini-mental state". A practical method for grading the cognitive state of patients for the clinician. J Psychiatr Res. (1975) 12:189–98. doi: 10.1016/0022-3956(75)90026-6 1202204

[130] Friedman NP Robbins TW. The role of prefrontal cortex in cognitive control and executive function. Neuropsychopharmacology. (2022) 47:72–89. doi: 10.1038/s41386-021-01132-0, PMID: 34408280 PMC8617292

[131] Shiffrin RM Schneider W. Automatic and controlled processing revisited. Psychol Rev. (1984) 91:269–76. 6571425

[132] Fabio RA Caprì T Romano M. From controlled to automatic processes and Back again: the role of contextual features. Eur J Psychol. (2019) 15:773–88. doi: 10.5964/ejop.v15i4.1746, PMID: 33680159 PMC7909205

[133] Loughrey DG Pakhomov SVS Lawlor BA. Altered verbal fluency processes in older adults with age-related hearing loss. Exp Gerontol. (2020) 130:110794. doi: 10.1016/j.exger.2019.110794, PMID: 31790801

[134] Diamond A. Executive functions. Annu Rev Psychol. (2013) 64:135–68. doi: 10.1146/annurev-psych-113011-143750, PMID: 23020641 PMC4084861

[135] Levinson SC Torreira F. Timing in turn-taking and its implications for processing models of language. Front Psychol. (2015) 6:731. doi: 10.3389/fpsyg.2015.00731 26124727 PMC4464110

[136] Campbell J Sharma A. Compensatory changes in cortical resource allocation in adults with hearing loss. Front Syst Neurosci. (2013) 7:71. doi: 10.3389/fnsys.2013.00071 24478637 PMC3905471

[137] Hornsby BW Kipp AM. Subjective ratings of fatigue and vigor in adults with hearing loss are driven by perceived hearing difficulties not degree of hearing loss. Ear Hear. (2016) 37:e1–e10. doi: 10.1097/AUD.0000000000000203, PMID: 26295606 PMC6681455

[138] Peelle JE. Listening effort: how the cognitive consequences of acoustic challenge are reflected in brain and behavior. Ear Hear. (2018) 39:204–14. doi: 10.1097/AUD.0000000000000494 28938250 PMC5821557

[139] Rosemann S Thiel CM. Neuroanatomical changes associated with age-related hearing loss and listening effort. Brain Struct Funct. (2020) 225:2689–700. doi: 10.1007/s00429-020-02148-w, PMID: 32960318 PMC7674350

[140] Ayasse ND Hodson AJ Wingfield A. The principle of least effort and comprehension of spoken sentences by younger and older adults. Front Psychol. (2021) 12:629464. doi: 10.3389/fpsyg.2021.629464, PMID: 33796047 PMC8007979

[141] Gabrys RL Tabri N Anisman H Matheson K. Cognitive control and flexibility in the context of stress and depressive symptoms: the cognitive control and flexibility questionnaire. Front Psychol. (2018) 9:2219. doi: 10.3389/fpsyg.2018.02219, PMID: 30510530 PMC6252356

[142] Sanchez K Dias JW Rosenblum LD. Experience with a talker can transfer across modalities to facilitate lipreading. Atten Percept Psychophys. (2013) 75:1359–65. doi: 10.3758/s13414-013-0534-x, PMID: 23955059 PMC3810281

[143] Bernstein JGW Venezia JH Grant KW. Auditory and auditory-visual frequency-band importance functions for consonant recognition. J Acoust Soc Am. (2020) 147:3712–27. doi: 10.1121/10.0001301, PMID: 32486805

[144] Bernstein LE Jordan N Auer ET Eberhardt SP. Lipreading: a review of its continuing importance for speech recognition with an acquired hearing loss and possibilities for effective training. Am J Audiol. (2022) 31:453–69. doi: 10.1044/2021_AJA-21-00112, PMID: 35316072 PMC9524756

[145] Deng J Zhou F Hou W Silver Z Wong CY Chang O. The prevalence of depression, anxiety, and sleep disturbances in COVID-19 patients: a meta-analysis. Ann N Y Acad Sci. (2021) 1486:90–111. doi: 10.1111/nyas.14506 33009668 PMC7675607

[146] Collaborators C-MD. Global prevalence and burden of depressive and anxiety disorders in 204 countries and territories in 2020 due to the COVID-19 pandemic. Lancet. (2021) 398:1700–12. doi: 10.1016/S0140-6736(21)02143-7 34634250 PMC8500697

[147] WHO. Mental Health and COVID-19: early evidence of the pandemic’s impact. Geneva, Switzerland: WHO (2022).

[148] Holube I von Gablenz P Bitzer J. Ecological momentary assessment in hearing research: current state, challenges, and future directions. Ear Hear. (2020) 41:79S–90S. doi: 10.1097/AUD.0000000000000934 33105262

[149] Bögels S. Neural correlates of turn-taking in the wild: response planning starts early in free interviews. Cognition. (2020) 203:104347. doi: 10.1016/j.cognition.2020.104347, PMID: 32563734

[150] Vandenbroucke JP von Elm E Altman DG Gøtzsche PC Mulrow CD Pocock SJ. Strengthening the reporting of observational studies in epidemiology (STROBE): explanation and elaboration. PLoS Med. (2007) 4:e297. doi: 10.1371/journal.pmed.0040297, PMID: 17941715 PMC2020496

